# Uncovering representations of sleep-associated hippocampal ensemble spike activity

**DOI:** 10.1038/srep32193

**Published:** 2016-08-30

**Authors:** Zhe Chen, Andres D. Grosmark, Hector Penagos, Matthew A. Wilson

**Affiliations:** 1 Department of Psychiatry, Department of Neuroscience & Physiology, New York University School of Medicine, New York, NY 10016, USA; 2 The Neuroscience Institute, New York University School of Medicine, New York, NY 10016, USA; 3 Department of Neuroscience, Columbia University Medical Center, New York, NY 10019, USA; 4 Picower Institute of Learning and Memory, Massachusetts Institute of Technology, Cambridge, MA 02139, USA

## Abstract

Pyramidal neurons in the rodent hippocampus exhibit spatial tuning during spatial navigation, and they are reactivated in specific temporal order during sharp-wave ripples observed in quiet wakefulness or slow wave sleep. However, analyzing representations of sleep-associated hippocampal ensemble spike activity remains a great challenge. In contrast to wake, during sleep there is a complete absence of animal behavior, and the ensemble spike activity is sparse (low occurrence) and fragmental in time. To examine important issues encountered in sleep data analysis, we constructed synthetic sleep-like hippocampal spike data (short epochs, sparse and sporadic firing, compressed timescale) for detailed investigations. Based upon two Bayesian population-decoding methods (one receptive field-based, and the other not), we systematically investigated their representation power and detection reliability. Notably, the receptive-field-free decoding method was found to be well-tuned for hippocampal ensemble spike data in slow wave sleep (SWS), even in the absence of prior behavioral measure or ground truth. Our results showed that in addition to the sample length, bin size, and firing rate, number of active hippocampal pyramidal neurons are critical for reliable representation of the space as well as for detection of spatiotemporal reactivated patterns in SWS or quiet wakefulness.

Sleep is critical to hippocampus-dependent memory consolidation^1^,^2^,^3^. Analyzing hippocampal ensemble spike data during both slow-wave sleep (SWS) and rapid-eye-movement (REM) sleep has been an important yet challenging research topic^4^,^5^,^6^,^7^,^8^,^9^,^10^. During awake active exploration, hippocampal pyramidal cells exhibit localized spatial tuning^11^. During sleep, in the absence of external sensory input or cues, the network is switched into a different state that engages in internally-driven computation. An important hallmark of sleep, the hippocampal sharp wave (SPW)-ripples, lasting between 50 to 400 milliseconds, is typically accompanied with an increased hippocampal network burst and population synchrony of pyramidal cells^1^. A central hypothesis is that the hippocampus and neocortex interact with each other during SPW-ripples^12^, and that hippocampal neurons fire such that the information transferred to the hippocampus during previous awake run behavior is reactivated at a fast timescale during SPW-ripple bursts, encoding information of spatial topology of familiar or novel environments, and goal-directed behavioral paths^10^,^13^,^14^,^15^,^16^,^17^,^18^,^19^. During run behavior, hippocampal place cells fire in sequences that span a few seconds as animals run through location-dependent receptive fields. During sleep, the same place cells fire in an orderly manner at a faster timescale within SPW-ripple events. While some sequences have been shown to reflect temporally-compressed spatial sequences corresponding to previous experiences by the rat^8^,^9^,^10^,^18^,^19^, the spatial content of a large fraction of SPW-ripple events remains unknown. Therefore, uncovering the neural representation of hippocampal ensemble spike activity or spatiotemporal firing patterns during sleep becomes critical for improving our understanding of the mechanism of memory consolidation and, in general, information processing during sleep.

To date, several statistical methods have been developed to analyze sleep-associated hippocampal ensemble spike activity, including pairwise correlation^4^,^5^, template matching^15^, sequence ranking^8^,^9^,^20^, and Bayesian population decoding^21^,^22^,^23^,^24^. A few observations of sleep data analysis are noteworthy. First, the SPW-bursts during sleep are sparse (low occurrence) and individual events are statistically independent. Second, the magnitude of neuronal population synchrony, measured as the spiking fraction of all recorded neurons during each network burst, follows a lognormal distribution: strongly synchronized events are interspersed irregularly among many medium and small-sized events^25^. Third, different brain states or experiences may induce changes in firing rate and firing timescale^15^,^26^,^27^. Fourth, there is no ground truth or behavioral measure. The pairwise correlation method ignores the spiking information at fine timescales and population synchrony; the template matching and sequence ranking is more sensitive to exact spike timing order and the number of active neurons. In contrast, Bayesian population decoding methods are more suited to tackle these issues in the presence of large neural ensembles^16^,^17^,^18^,^23^. However, to our knowledge, there is no precedent for a systematic investigation of these issues using any of these methods.

In this work, we investigate these important statistical issues in greater detail by applying two neural population decoding methods to rat hippocampal ensemble spike data recorded in different states. One decoding method is based on topographic or receptive field representations^21^,^22^, while the other is based on topological representation without *a priori* measure of place receptive fields^28^,^29^,^30^. We first create “synthetic” sleep data by binning and resampling spike trains obtained during active locomotion to simulate important factors that characterize SPW-ripple events, and then compare the resulting decoded spatial representations to the animal’s actual run trajectory. This allows us to test two important questions of hippocampal population codes related to sleep and memory replay: *representation power* (“how reliably is the spatial environment represented?”) and *detection power* (“how can one detect significant spatial or behavioral state sequences?”). We use rat hippocampal ensemble recordings in two- and one-dimensional spaces to investigate these questions separately, and we further compare the performance of topographic vs. topological representation-based decoding methods to SPW-ripple associated spike data.

## Results

### Data

We analyzed five datasets (Table 1) derived from experimental hippocampal ensemble spike data, recorded from multiple Long-Evans rats under different environments, behaviors and brain states. The animals’ behavioral trajectories from Datasets 1 to 4a are shown in Supplementary Fig. 1. To analyze rat hippocampal ensemble spike data, we considered two model-based Bayesian decoding methods based on different statistical assumptions (*Methods*, Supplementary Fig. 2). One decoding method is based on topographic or receptive field representations (termed Decode_wRF_—population decoding method using neuronal receptive fields, see Supplementary Fig. 3). The other is based on topological representation that aims to discover latent structures of sequential or spatiotemporal pattern of activity of cells without the assumption of behavioral measures (termed Decode_woRF_—population decoding method without using neuronal receptive fields). The first method is supervised in that it requires training data for constructing place receptive fields in the encoding phase. The second method is purely unsupervised, which is developed based on an *m*-state hidden Markov model (HMM), with an inherent *m* × *m* state-transition matrix ***P***.

Sleep-associated hippocampal ensemble spike data are characterized by several important features: (1) shorter epochs (separated by periods of non- or low-spike activity); (2) small active cell ratio within each epoch; (3) different timescales from behavior. One fundamental assumption is that many sleep-associated hippocampal ensemble spikes preserve the order of temporal firing sequences experienced in behavior. In the following analyses, we first created “synthetic” sleep-like hippocampal ensemble spike data (derived from awake run behavior) and systematically investigated the issues of the length of data epochs, the number of participated neurons, temporal bin size and spike rate. The use of synthetic data allowed us to quantitatively assess the *representation power* (or decoding accuracy) in hippocampal ensemble representations. We then extended the analyses to experimental sleep data in complete absence of behavior measure and assessed the question of *detection power*. All reported statistics are shown in mean ± SEM.

We used two established criteria for quantitative assessment: one is the decoding error with respect to the animal’s position, and the other is the weighted correlation^17^,^18^ and the associated Z-score or equivalent Monte Carlo *P*-value of detected significant replay events (*Methods*). The first criterion, which assesses the representation power (i.e., how does the population spike activity reliably represents the environment, ref. 29), was tested on two-dimensional environments (Datasets 1 and 2, see an illustration in Supplementary Fig. 4). The second criterion assesses the detectability issue (Datasets 3, 4a and 4b, ref. 31).

### Impact of random splitting

Unlike awake behavior, hippocampal neuronal populations fire in a sporadic manner during sleep, either within or outside the period of SPW-ripples. During awake run behavior, rat hippocampal ensemble spike data were binned with a temporal bin size of Δ = 250 ms into *T* discrete bins (i.e., *T*Δ corresponds to total recording time). We applied a speed filter of 15 cm s^−1^ to exclude immobile periods. As a first step to create sleep-like data structure (Supplementary Fig. 5A), we evenly split the run-associated ensemble spike into 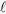 epochs. Each epoch was comprised of 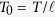 bins per epoch (bpe) and provided an independent measurement for further statistical analysis. Within each epoch, the temporal order of spiking sequences within cell assembly was preserved or reversed (with equal probability 0.5). The special case when 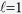 and *T*_0_ = *T* bpe corresponds to the run-associated spike data; when *T*_0_ = 1 bpe, all spike bins are independent. Generally, the greater the *T*_0_ value, the more temporal information is available within each epoch (which are used to infer the state-transition matrix ***P*** in Decode_woRF_). In analogy to sleep, *T*_0_ = 10 bpe roughly reflects the typical number of temporal bins of 200-ms hippocampal ripple-associated spike data with 20 ms bin size.

**Figure 1:**
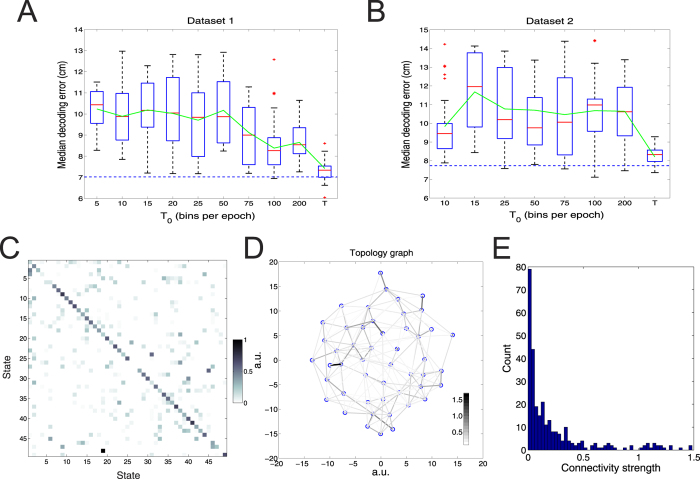
Illustration and decoding performance of population decoding methods. (**A**,**B**) Box plots of median decoding error from Decode_woRF_ with varying values of *T*_0_ (bins per epoch), for Datasets 1 and 2, respectively. The green curves are the averaged median decoding error. The median decoding error of Decode_wRF_ was independent of *T*_0_ (horizontal dashed line; 7.02 for Dataset 1, 7.73 for Dataset 2). Representative examples of inferred state-transition matrix (**C**) from Decode_woRF_ and the derived topology graph (**D**) from Dataset 2 (dark color represents high connectivity strength). The percentage of nonzero entries in (**C**) is 14.8%. (**E**) Histogram of nonzero connectivity strengths (*P*_*ij*_ + *P*_*ji*_, *i* ≠ *j*) for panel (**C**) (mean: 0.23; SD: 0.32).

Using all available neuronal ensemble spike activities from Datasets 1 and 2, we systematically varied *T*_0_ and computed the median decoding error (mean ± SEM). At each *T*_0_ configuration, analyses were repeated *n* = 50 independent Monte Carlo runs, with each run encountering different realization of simulated data. Assuming no temporal prior, the decoding performance of Decode_wRF_ remained unchanged for varying *T*_0_ (horizontal dashed line, Fig. 1A,B). This is because the receptive filled is computed based upon the average spike activity over the entire or part of the behavioral episode. Once the receptive field is identified and the likelihood model is fixed, the temporal information becomes irrelevant for estimating the position at each temporal bin. In contrast, the population representation capacity and decoding accuracy of Decode_woRF_ changed as a function of *T*_0_. Our analysis suggested that the mean decoding error (green curves, Fig. 1A,B) was relatively stable with varying *T*_0_ < *T*, but the result variability within the same *T*_0_ configuration was relatively high (except for *T*_0_ = *T* bpe). The source of variability was contributed by at least two factors: First, because of random data splitting, breaking the temporal relationship in a spike train also destroy the spatial-temporal relationship (i.e., spike patterns with respect to animal’s run behavior during those periods). For instance, a given position is associated with different spike patterns that depend on the actual trajectory leading to it, such as animal’s heading, speed, and previous location. Second, the intrinsic Monte Carlo optimization nature of Decode_woRF_ induces additional variance (e.g., slow convergence of Markov chains)^30^.

The inferred number of states *m* derived from Decode_woRF_ was relatively stable (*m* ∈ [33, 37] for Dataset 1; *m* ∈ [46, 53] for Dataset 2). As a qualitative assessment, we transformed and depicted the matrix ***P*** (Fig. 1C) via a topology graph (Fig. 1D), which describes the connectivity between the state (“spatial location”) and the topological representation of the environment^28^,^29^. The topology graph is in arbitrary unit (a.u.): each note represents a state or virtual location, and the strength between two nodes indicates the pairwise connectivity (*P*_*ij*_ + *P*_*ji*_, with dark color representing high strength). We also assessed the distribution of connectivity strengths and associated statistics (Fig. 1E). A detailed examination of the inferred 49 × 49 matrix ***P*** showed that the majority of nodes had more than one pair of significant nonzero connectivity. For instance, if we used a conservative high connectivity strength threshold 0.2–60% percentile of the empirical distribution, then 44/49 nodes had at least two connected nodes, whereas nearly half (24/49) of nodes had between 3 and 5 connected nodes. Combining the quantitative assessment and qualitative visualization, we reached the interpretation that the topology graph in Fig. 1D resembles a two-dimensional grid; its shape was invariant to the permutation of states in ***P***. Although the exact values of ***P*** might be quantitatively different in random Monte Carlo simulations, the derived two-dimensional topology graphs were qualitatively similar with respect to with varying *T*_0_ configurations (data not shown) and varying subsets of neurons^29^.

### Impact of the number of cells

Compared to awake experiences, firing rates of hippocampal neurons during post-run sleep episodes were reduced but highly correlated^15^,^25^. However, the participation of the active hippocampal cells during sleep can be highly variable. More importantly, only a small subset of pyramidal neurons are active during individual SPW-ripple events^15^. To simulate such conditions, we used a fixed value of *T*_0_ = 10 bpe and randomly sampled a subset of cells from the neuronal population (*ρ* = 30–100%, with a minimum of 10 cells being active); only those selected neurons were used in subsequent decoding analyses.

**Figure 2:**
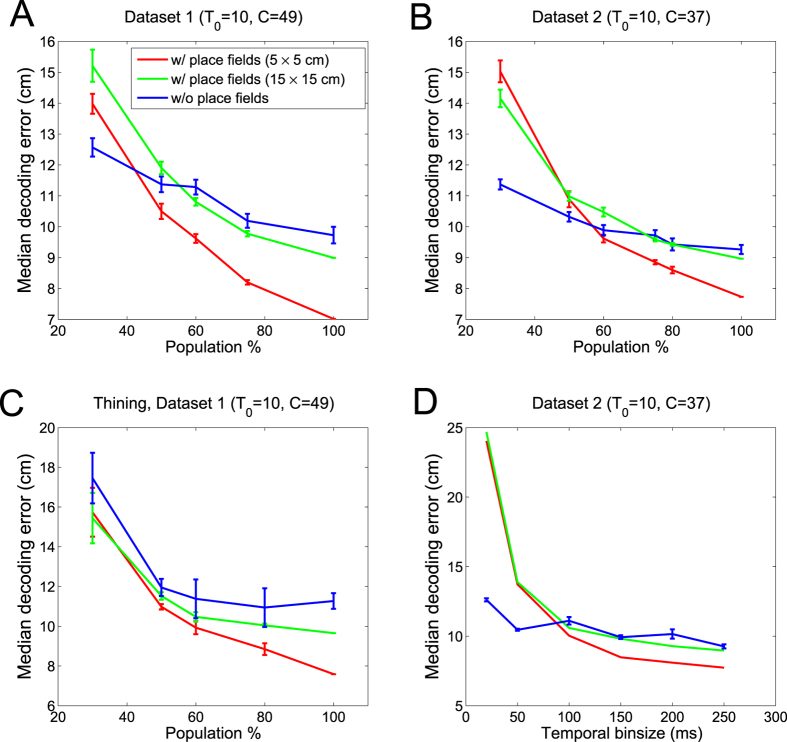
Comparison of median decoding error between Decode_wRF_ and Decode_woRF_. (**A,B**) Decoding error decreased with increasing numbers of cells in neuronal population. (**C**) Decoding error changed with respect to varying fractions of active neurons (under thinning) and (**D**) changed with respect to varying temporal bin size. Error bar denotes SEM (*n* = 50 Monte Carlo runs).

We found that the decoding error monotonically decreased as the increasing fraction of active neurons (Fig. 2A,B; see also the evolution of error distribution in Supplementary Fig. 6). When the number of cells fell below a certain percentage (~50%), Decode_woRF_ outperformed Decode_wRF_, yet the exact statistics varied between the two tested datasets. The slope of error curve in Decode_woRF_ was flatter, consistent with our previous finding that the topology-based coding may be more robust for spatial representation^29^. This is possibly because the Decode_woRF_ does not require a precise receptive field representation; in contrast, Decode_wRF_ method is more dependent on the neurons that have a well-described place receptive field representation; when the receptive field characterization is less accurate due to the finite sampling issue, it may produce a large error.

**Figure 3:**
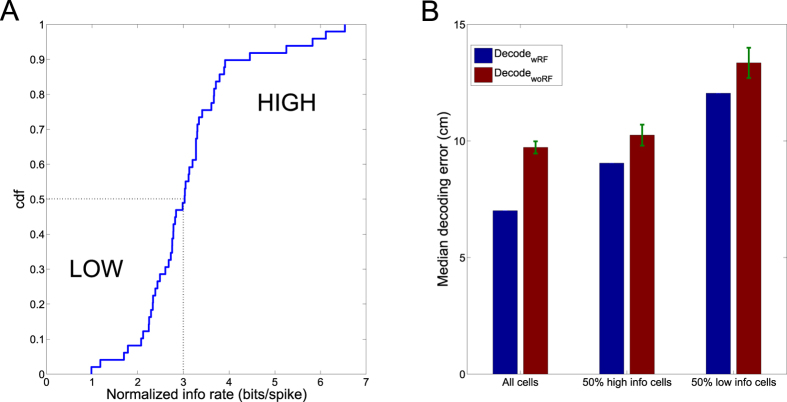
Specificity of hippocampal neurons in cell population on the decoding error. (**A**) Cumulative distribution of normalized spatial information rate (bits/spike) of 49 hippocampal neurons (Dataset 1). (**B**) Comparison of median decoding error by using spatial-information high vs. low subpopulations (*T*_0_ = 10 bpe, *ρ* = 0.5; error bar denotes SEM, *n* = 50 Monte Carlo runs).

To test specific relationship between population representation and the cell physiological properties, we evenly split the neurons of Dataset 1 into two groups (upper vs. lower 50% percentile) according to their normalized spatial-information rates (*Methods*, Fig. 3A). Under the same configuration (*T*_0_ = 10, *ρ* = 50%), we compared the decoding accuracy of two population methods based on Monte Carlo simulations. The result (Fig. 3B) indicated that the information-rich neuron subpopulation had a greater influence on representation or decoding accuracy (*P* < 0.001, Wilcoxon signed rank test).

In experimental sleep recordings, different subsets of neurons often fire at individual, isolated sleep episodes. To simulate this situation, we introduced additional level of randomness by assuming that distinct neuronal subpopulations (but with identical ratio *ρ*) are randomly active at individual epochs—this was in direct contrast to the previous assumption that the same subpopulations were engaged in all episodes. As a demonstration, we fixed *T*_0_ = 100 and applied Decode_woRF_ to Dataset 1. As expected, the decoding accuracy further degraded: for *ρ* = 0.8, 0.7, 0.6, 0.5, 0.4, 0.3, the median errors were 9.07 ± 0.18, 10.02 ± 0.14, 10.25 ± 0.13, 11.51 ± 0.12, 11.99 ± 0.12, 12.53 ± 0.16 cm (*n* = 50 Monte Carlo runs), respectively. The error was not only greater than the error in the case of *ρ* = 1 (8.51 ± 0.18 cm, Fig. 1B), but also greater than the error with fixed subpopulations (*T*_0_ = 100 vs. *T*_0_ = 100* bpe, Table 2).

### Impact of bin size, spike rate and conjunctive factors

During different sleep stages, hippocampal neurons fire at different timescales^15^,^23^. To examine the influence of temporal bin size, we fixed *ρ* = 1 and *T*_0_ = 10 bpe, and varied bin size Δ (20, 50, 100, 150, 200, 250 ms) to repeat the decoding analysis. Note that a decreasing Δ would increase the number of discrete bins *T*. For Decode_wRF_, the decoding accuracy reduced with a decreasing Δ. This might be due to violation of Poisson assumption while using a small bin size or due to the presence of theta sequences (i.e., the decoded position may be systematically ahead of actual animal’s position). In contrast, the decoding performance of Decode_woRF_ (blue curve, Fig. 2D) was relatively stable for various Δ, possibly because its Bayesian inference procedure is less sensitive to the Poisson firing assumption^30^.

Next, we thinned the spike train data by downsampling such that there was no more than one spike per time bin, which was aimed to simulate the sparse spiking in a finer timescale during sleep. As a result, the instantaneous firing rate reduced to 25–50% of the original rate. We found that the spike thinning procedure further degraded the decoding performance, and the decoding accuracy also dropped with decreasing number of neurons (Fig. 2C vs. Fig. 2A).

Lastly, we jointly varied two parameters (such as *T*_0_ and *ρ*, or *T*_0_ and Δ), and repeated the decoding analysis. As shown in Table 2, we obtained consistent findings as in Fig. 2 (see also Supplementary Fig. 7): (1) For fixed Δ and *ρ*, there was a decreasing trend in decoding error with increasing *T*_0_, but the performance was relatively stable; (2) Regardless of *T*_0_, decoding error decreased with increasing *ρ*; (3) For fixed *T*_0_, there was a decreasing trend in decoding error with increasing Δ.

### Impact of non-place cells

Next, we investigated if and how the presence of non-place cells would affect the decoding accuracy. A non-place cell implies that the putative pyramidal cell is not significantly modulated by spatial location, or its spatial tuning curve is flat. A high ratio of non-place cells implies a low signal-to-noise ratio (SNR) for fixed number of cell population. To simulate such a condition, we randomly selected a small subset of place cells (Dataset 1, many of which have overlapping place fields, see Supplementary Fig. 3) and evenly distributed spikes in time proportional to animal’s space occupancy (such that their average firing rates remained unchanged). Under the same configuration (*T*_0_ = 100 bpe), we found the decoding error of two methods increased with growing number of non-place cells (Supplementary Fig. 8). At first, Decode_woRF_ was slightly worse than Decode_wRF_, but the gap gradually reduced with decreasing SNR (Supplementary Fig. 8A, red vs. blue solid lines); and Decode_woRF_ outperformed Decode_wRF_ significantly (*P* = 6 × 10^−5^, Wilcoxon signed rank test) in the worst scenario. This result confirmed the robustness of Decode_woRF_ under a low SNR.

### Significance testing via randomly shuffled data

We tested our population decoding methods by comparing their estimate statistics derived from experimental data with those derived from randomly shuffled data (Supplementary Fig. 5B). Specifically, we used the hippocampal ensemble spike activity collected during animal’s run behavior (speed >15 cm s^−1^) in a circular track environment (Dataset 3). Upon completion of unsupervised learning (Decode_woRF_), we recovered the state trajectory, which correlated with the animal’s run trajectory (Pearson’s correlation ∈ [0.73, 0.79] derived from 10 Monte Carlo runs, *P* = 1.5 × 10^−15^, Supplementary Fig. 9A). In addition, we obtained the state transition matrix and state field matrix, which were both qualitatively similar to the behavior-derived ground truth (Supplementary Fig. 9B,C). The average *maximum a posteriori* (MAP) probability score derived from Decode_woRF_ was 0.8814, and the weighted correlation was 0.8848. These statistics were also similar to those from Decode_wRF_, except that Decode_wRF_ required receptive fields or behavioral measure *a priori*.

We further constructed 1000 shuffled datasets. Each randomly shuffled dataset was subject to both temporal bin and cell identity shuffles (*Methods*). The Monte Carlo weighted correlation *R* and average MAP probability scores derived from the shuffled data than those derived from the raw data were significantly lower (Monte Carlo *P* < 10^−7^, Supplementary Fig. 10). These results demonstrated that, in the absence of behavioral measures (therefore no decoding error can be computed), theses metrics can be used as quantitative measures to assess the quality of reconstructed event for detection purpose. In the remaining analyses, we used *R* and its associated Z-score (or equivalent Monte Carlo *P*-value) for assessment.

**Figure 4:**
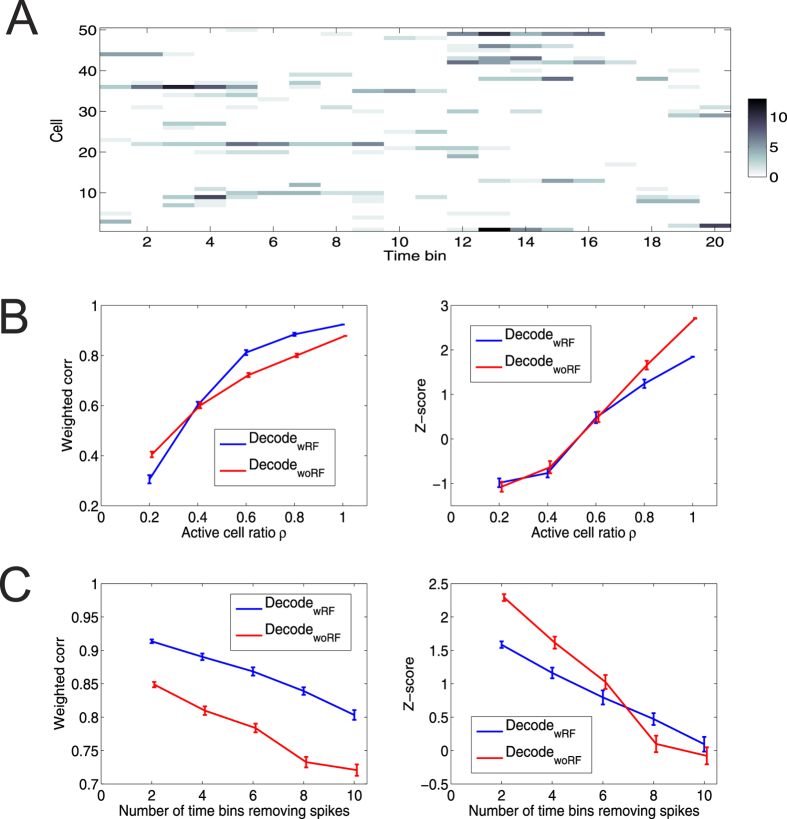
Comparison of detection reliability between Decode_wRF_ and Decode_woRF_. (**A**) Segment of a spike count matrix with 20 temporal bins. (**B,C**) Weighted correlation (*Left*) and Z-score (*Right*) for varying active cell ratio *ρ* (**B**) and for removing spikes across different number of bins (**C**). Error bar denotes SEM (*n* = 50 Monte Carlo runs).

To compare the detection reliability and specificity between Decode_wRF_ and Decode_woRF_, we selected a random segment of run trajectory (*T*_0_ = 20 bpe, Fig. 4A), and systematically manipulated the ensemble spike activity during that time interval as follows: (1) We randomly removed 20–80% of cells from the population (i.e., *ρ* = 0.2–0.8). (2) Using all active cells (*ρ* = 1), we randomly removed spikes in selected temporal bins from each cell, with the number of bins ranging from 2 to 10 (i.e., 10–50% of *T*_0_)—which would sparsify and remove certain temporal structures in the ensemble spikes. We simulated each condition with 100 Monte Carlo runs, and each run produced an independent test set. We applied Decode_wRF_ and Decode_woRF_ to those test sets and computed their *R* and Z-scores. The result comparison is shown in Fig. 4 (see also Supplementary Fig. 11 for scatterplot comparison). As the number of active cells dropped, the detection power of both methods decreased accordingly (Fig. 4B). In this specific example, the |*R*| value was below 0.5 when *ρ* = 0.2 (i.e., 10 cells). In terms of the Z-score, majority of simulated events were non-significant when *ρ* < 0.8. Removing spikes also degraded the detection power (Fig. 3C; see also Supplementary Fig. 12). Together, these results suggest that the detection power of Decode_woRF_ was more favorable in those tested conditions.

### Analysis of ripple-associated spike data in quiet wakefulness

We also tested our methods on ripple-associated hippocampal ensemble spike data during quiet wakefulness (QW)—the awake brain state involved in memory replay similar to SWS^16^,^17^,^18^,^19^,^23^,^32^,^33^. In a long recording (Datasets 4a and 4b), hippocampal ensemble spikes were collected in the 4-hr pre-run and 4-hr post-run periods (inside a rest box in a familiar environment), separated by 40-min run period on a circular track in a novel setting (see Supplementary Fig. 13 for brain state classification). From Dataset 4b, we identified off-the-track candidate events based on hippocampal local field potential (LFP) and multi-unit activity (*Methods*), and further excluded the epochs with low fraction of active cells (<10%). See Table 3 for the summary statistics of candidate events in different states.

**Figure 5:**
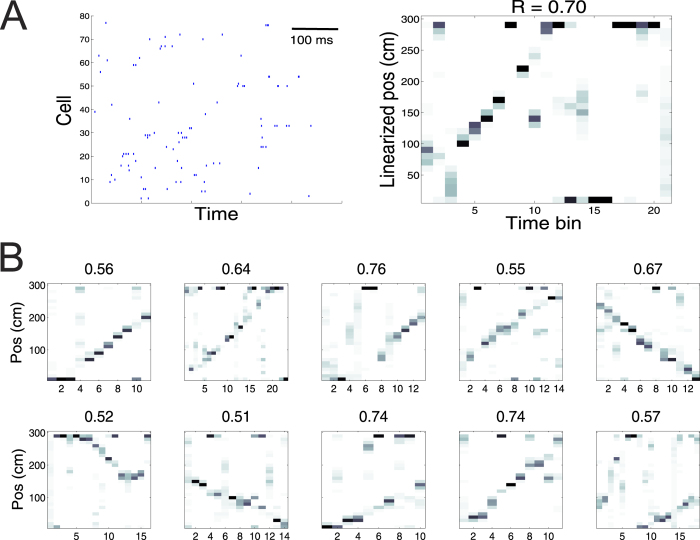
QW-associated ensemble spike data analysis. (**A**) Example of spike rasters and the associated decoded spatial trajectories in quiet wakefulness. The number at the top of each panel indicates the absolute weighted correlation |*R*|. (**B**) Examples of detected significant replays. X-axis represents time bin (bin size Δ = 20 ms).

The ratio of active cells across all selected epochs was *ρ* = 0.181 ± 0.002 (maximum 0.68, median 0.16). We binned each epoch with Δ = 20 ms, resulting in *T*_0_ = 11.5 ± 0.2 bpe (maximum 39, median 10). We then reconstructed the spatial (or state) trajectory based on the place field (or state field) *λ*_*c*_(***S***) (where the state field was inferred by Decode_woRF_ from the run-associated ensemble spikes alone). For each epoch, we computed the weighted correlation *R* and its associated Z-score, and compared them with those obtained from randomly shuffled data. Figure 5 shows some examples of detected significant replays during post-QW epochs. Qualitative and quantitative assessment of those replay events indicated diverse (forward vs. reverse) spatiotemporal structures.

### Analysis of SWS-associated spike data

At last, we applied our population decoding methods to experimental SWS-associated hippocampal ensemble spike activity (Dataset 4b). The candidate events with >10% active cells were selected for analysis (Table 3), and each event was treated as an independent epoch.

**Figure 6:**
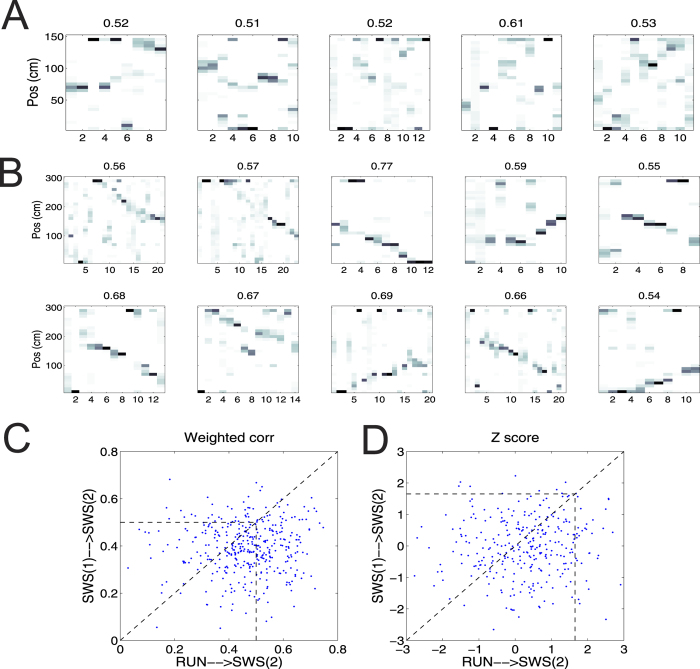
SWS-associated ensemble spike data analysis. (**A,B**) Examples of detected significant pre-SWS (**A**) and post-SWS (**B**) reactivation events. The number at the top of each panel indicates |*R*|. X-axis represents time bin (bin size Δ = 20 ms). (**C,D**) Testing predictability of RUN and SWS(1) data for SWS(2): scatterplot comparison of weighted correlation (**C**) and Z-score (**D**) between RUN → SWS(2) and SWS(1) → SWS(2). Lower left corner marked by dashed line indicates the non-significance zone.

Specifically, there was no difference in *ρ* between pre- and post-SWS (*P* = 0.31, rank-sum test; pre-SWS: *ρ* = 0.175 ± 0.003, maximum 0.45, median 0.16; post-SWS: *ρ* = 0.178 ± 0.003, maximum 0.53, median 0.16). With Δ = 20 ms, the number of bins per epoch was slightly longer in pre-SWS than in post-SWS epochs for Dataset 4b (*P* = 0.006, rank-sum test; pre-SWS: *T*_0_ = 12.7 ± 0.3 bpe; post-SWS: *T*_0_ = 11.9 ± 0.2 bpe). Hippocampal neurons’ mean firing rate remained stable between pre-SWS and wake as well as between post-SWS and wake (Supplementary Fig. 14), although the mean firing rate in wake was significantly higher (Wilcoxon signed rank test, *P* < 1.3 × 10^−5^). To examine significant pre- and post-SWS reactivation events, we used the inferred *λ*_*c*_(***S***) during RUN to estimate the state trajectory and posterior probability scores of candidate events during respective pre- and post-SWS periods. Some detected reactivation examples are shown in Fig. 6A,B, respectively. In comparison, the quality of detected post-SWS replay events was qualitatively better in terms of trajectory continuity than that of detected pre-SWS events. We identified statistically significant events based on their computed *R* and Z-score statistics (Table 3). The absolute number and the ratio of significant events increased from pre-SWS to post-SWS. In addition, the Z-score among the significant events was greater in post-SWS (*P* < 0.01, rank-sum test). These results suggested that the neuronal ensemble patterns shared more similar structures between post-SWS and RUN than between pre-SWS and RUN—a finding consistent with the pairwise correlation method (ref. 4, see Supplementary Fig. 15) and another independent investigation^31^.

We further examined the nonstationarity of sleep epochs by comparing the results derived from the first and second-half of post-SWS candidate events (i.e., SWS(1) and SWS(2) have the same epoch number that had no less than 10% active cells, defined by 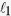 in Table 3). For Decode_woRF_, we found that the *T*_0_, *ρ* and *R* statistics were similar between SWS(1) and SWS(2), but the numbers that aim to assess the significance of detected events (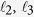 and 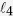 in Table 3) all decreased in SWS(2). This could be due to the fact that memory reactivation was more frequent in SWS(1) than in SWS(2), or the representation power decreased in SWS(2). To test the predictive power of SWS(1) to SWS(2), we applied Decode_woRF_ to SWS(1) and inferred the SWS-state field *λ*^*SWS*^ (which was distinct from 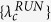 estimated from spikes alone in run behavior). We then used 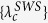 to assess the *R* statistic for SWS(2), and compared that with the *R* statistic obtained from 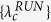. A scatterplot comparison (Fig. 6C,D) showed a decrease trend in |*R*| (*P* = 10^−15^) and Z-score (*P* = 1.1 × 10^−4^, both Wilcoxon signed rank test) from using 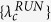 to using 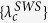, suggesting a reduction of predictive power in SWS(1) → SWS(2).

## Discussion

Interrogating the temporal structure and content of sleep-associated hippocampal ensemble spikes can reveal important mechanisms of hippocampal sequence generation^34^,^35^,^36^ or diverse contributing roles of hippocampal neurons in plasticity^31^. However, analysis of such spike data has posed a great challenge. In this study, we applied two population decoding methods (Decode_wRF_ and Decode_woRF_) to rat hippocampal ensemble spikes recorded in different brain states, aiming to infer the animal’s actual or virtual spatial location based on their spatiotemporal firing patterns. In terms of representation and detection power, population decoding methods are more powerful than the conventional correlation or sequence methods for discovering inherent structures of the ensemble spike data. Moreover, since the latent state corresponds to an abstract or virtual behavioral correlate in Decode_woRF_, detecting statistical significance of temporal sequences is not restricted by the line fitting procedure^23^, which may become an issue for Decode_wRF_ in the presence of cursive trajectories (e.g., in a two-dimensional environment) or in the presence of discontinuity in spatial trajectory (see an example in Supplementary Fig. 16). Moreover, our Bayesian inference procedure automatically identifies the model order in Decode_woRF_ to allow optimal choice of spatial resolution given observed ensemble spikes. From the analyses of both synthetic and experimental data, we found that the representation and detection power of both population decoding methods were strongly dependent on the number of active place cells. Since place cells did not contributed evenly in representation (Fig. 3 and Supplementary Fig. 6), fast-firing neurons did not always contain the most spatial information (bits/spike). In fact, recent findings suggested that slow-firing neurons may contribute more to neuronal sequences from pre to post-sleep^31^. Considering the low fraction of active hippocampal cells in sleep and the lognormal distributed phenomenon^25^, a large number of recorded place cells are necessary to secure the statistical power for sleep data analysis.

Population decoding methods have been proven useful in studying information transmission and sensory coding of neural systems^37^,^38^. Here, our model-based decoding approach offers a statistical framework to assess the content of sleep-associated hippocampal ensemble spikes, which may reveal important mechanism insights on hippocampal neurons in memory consolidation. Similar to other reports^18^,^31^, we found that the reactivated spatial trajectories or sequences in hippocampal ensemble representations were better correlated and more sharply defined in post-SWS than in pre-SWS. Nevertheless, several statistical questions still remain unanswered. One puzzle is how can we extract significant non-spatial information encoded in sleep? Another pressing issue is to design statistical methods that can adapt to specific temporal (e.g., inhomogeneous, nonstationary, and heteroscedastic) structure of ensemble spike data. Thus far, we have used a uniform temporal bin size throughout SWS, yet finding the optimal timescale is critical for decoding analyses. Our current study has focused on hippocampal ripple-associated ensemble spike activity and ignored other spike activities outside of ripples. Analyzing *continuous* sleep-state spike activity would be the next goal. Notably, hippocampal and cortical neurons operate at a different timescale in REM sleep from SWS. The question of interpreting sparse and sporadic REM-associated hippocampal spike activity remains unresolved. A recent report has revealed similar geometric structure in neural correlations of hippocampal neurons between active navigation and REM sleep^39^. It would be interesting to test the population decoding methods on such independent recordings. In addition, these methods can be tested to evaluate brain state transition.

In principle, our unsupervised population decoding framework can be applied to hippocampal-cortical or thalamocortical ensemble spikes in sleep^10^,^40^,^41^,^42^. Joint investigation of spatiotemporal sequences in these circuits during sleep replay events are crucial to infer the communications and information transfer between these circuits during memory consolidation. Given a large neuronal ensemble, the Decode_woRF_ method is appealing since it requires no explicit measure of behavior or receptive fields, where the latent states may represent non-spatial features of experiences or distinct behavioral patterns that cannot be measured directly. Ultimately, it is critical to discover nonlinear interactions and extract spatiotemporal organization among neuronal ensembles, integration of such principles and data-driven neuronal models will be the key to revealing intrinsic structures of neuronal ensemble spikes.

## Methods

### Animal behavior and neurophysiological recordings

Long-Evans rats were freely foraging in familiar spatial environments for a period of 30–45 minutes (Datasets 1–3). In Datasets 4a and 4b, rats were first put in a sleep box of a familiar environment for 4 hours, and then moved to a circular track (novel environment) for running about 45 minutes, and then put back to the sleep box for another 4 hours (ref. 31). All procedures were approved by the MIT and NYU Institutional Animal Care and Use Committee and carried out in accordance with the approved guidelines.

Custom microelectrode drive (Datasets 1–3) or silicon probe arrays (Datasets 4a and 4b) were implanted unilaterally or bilaterally in the animal’s dorsal hippocampal CA1 area. Spikes were acquired with a sampling rate of 31.25 kHz and filter settings of 300 Hz–6 kHz. Two infrared diodes alternating at 60 Hz were attached to the drive of each animal for position tracking. We used a custom manual clustering program for spike sorting to obtain well-isolated single units. Details are referred to previous publications^23^,^31^. Putative interneurons were identified based on the spike waveform width and average mean firing rate. In addition, all putative pyramidal neurons selected for analysis had peak firing rate >1 Hz.

### Bayesian decoding

The Bayesian decoding algorithms is formulated within a state-space model framework^21^,^22^,^28^,^29^,^30^. Let *S*_*t*_ represent the animal’s spatial position label at discrete time *t*, and let ***y***_*t*_ represent the observed neuronal population spike count between ((*t* − 1)Δ, *t*Δ], where Δ is the temporal bin size. The state variable *S*_*t*_ is assumed to follow a first-order Markovian dynamics and characterized by *p*(*S*_*t*_|*S*_*t* − 1_). The goal of Bayesian decoding is to infer the posterior probability *p*(*S*_*t*_|***y***_1:*t*_) given all the spike history up to time *t*. Here we assumed that conditional on the state *S*_*t*_, the population firing of *C* hippocampal place cells follows a Poisson likelihood model


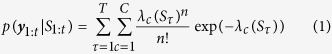


where 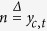 denotes the spike count from the *c*-th neuron at the *τ*-th temporal bin. In light of Bayes’ rule, the posterior distribution of the state *S*_*t*_ is given by


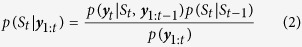


where *p*(*S*_*t*_|*S*_*t*−1_) denotes the temporal prior and *p*(***y***_1:*t*_) is a normalizing constant.

For decoding analysis, we used two population decoding methods. In the first method (Decode_wRF_, Supplementary Fig. 2A), the animal’s spatial position was measured during run behavior, which was further used to estimate neuronal receptive fields *λ*_*c*_(***S***) (note: ***S*** is continuous-valued and can be finite or infinite, with proper dimensionality depending on the spatial environment). Hippocampal place fields were estimated using a spatial bin size of 10 cm for one-dimensional tracks, and bin size of 5 × 5 cm^2^ or 15 × 15 cm^2^ for two-dimensional space, and further smoothed using a Gaussian template (5 × 1 for one-dimensional or 3 × 3 for two-dimensional environment) with a half SD. This method consists of both encoding and decoding phases, where the encoding phase is supervised.

In the second method (Decode_woRF_, Supplementary Fig. 2B), the animal’s behavioral measures are assumed inaccessible, therefore no place fields can be estimated from the behavioral data. The second method only consists of decoding phase, and it is purely unsupervised. In this case, *S*_*t*_ represents a discrete-state label for the spatial position, and it can be either finite or infinite depending on the statistical assumption, spatial resolution, and the size of data. In this special case, the state space model is a hidden Markov model (HMM); trajectories across spatial locations (“states”) were associated with consistent hippocampal ensemble spiking patterns, which were characterized by a stationary state transition matrix defining *p*(*S*_*t*_|*S*_*t*−1_) (e.g., Fig. 1C). The observed spike count data was defined by a Poisson probability distribution *p*(***y***_*t*_|*S*_*t*_) in equation (1). Unlike Decode_wRF_, the state of Decode_woRF_ was subject to permutation ambiguity due to the lack of behavior measure. The goal of inference is to estimate the *maximum a posteriori* (MAP) state sequences *S*_1:*T*_ and the unknown state transition matrix and rate parameters *λ*_*c*_(***S***) with respect to the state space ***S*** = {*S*_*i*_} (where *S*_*i*_ ∈ {1, 2, …, *m*} are categorical variables). See refs 28, 29, 30 for details of model description and inference procedure. Briefly, first, we applied a Bayesian nonparametric version of the HMM: hierarchical Dirichlet process (HDP)-HMM, combined with advanced Markov chain Monte Carlo (MCMC) inference methods^30^. The number of latent states, *m*, was automatically inferred from the MCMC inference procedure (Supplementary Fig. 17A). Second, we constructed a “state space map” between the discrete state and the spatial position (see Supplementary Fig. 17B for illustrations). For one-dimensional environment, the ideal state space map shall have a one-to-one mapping. Third, to visualize the inferred state transition matrix (Fig. 1C), we applied a force-based algorithm to derive a scale-invariant topology graph that defines the connectivity between different states (nodes) (Fig. 1D), which provided intuitive result interpretation and qualitative assessment.

In the testing phase, two population decoding methods were operated in a similar way, except with different *λ*_*c*_(***S***) (one constructed from behavior and the other estimated from spikes alone). We applied these two methods to reconstruct the spatial position or state *S* at each time bin, and computed the average MAP probability score from multiple bins.

### Information rate of hippocampal neurons

Information-theoretic measures have been used to characterize the information of hippocampal neurons^43^. We define the spatial information rate of the *c*-th hippocampal neuron as follows


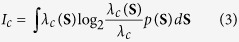


where *λ*_*c*_(***S***) denotes the mean firing rate at spatial location ***S***, and *λ*_*c*_ = ∫*λ*_*c*_(***S***)*p*(***S***)*d**S*** denotes the total average firing rate (spikes/s). The unit of *I*_*c*_ is measured by bits/s. To account for the total firing rate effect, we compute the *normalized* information rate, 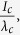 measured by bits/spike.

### Statistical assessment

For Decode_wRF_, we computed the median decoding error between the estimated animal’s position and the actual position. For Decode_woRF_, the animal’s actual position was solely used for result assessment. Based on the state space map, we estimated animals spatial trajectories and computed the median decoding error^29^,^30^.

Statistically significant reactivation events were determined by three established criteria^17^,^18^: (1) The absolute “weighted correlation” *R* (which measures the strength of correlation between the changes in probability values across time and spatial position) greater than 0.5. (2) The time length is greater than five temporal bins (i.e., 100 ms for QW or SWS epochs). In addition, the MAP probability score equal or less than the threshold (5/total number of position bins; below which is just a chance level) is also regarded non-significant. In addition, we generated shuffled candidate events from each pre-identified candidate event, and computed the *R*_shuffle_ from randomly shuffled population spike data. Two types of shuffling operations were considered: *temporal shuffling* and *cell shuffling*. Algebraically, the spike count matrix was subject to both row (temporal) and column (cell) shuffle operations. A total of 1000 shuffled samples were constructed. From the raw and shuffled statistics, we computed the Z-score for *R* as follows^31^: *Z* = (R − mean of R_shuffle_)/(SD of R_shuffle_). (3) The Z-score of *R* is greater than 1.65 (equivalent to one-side *P*-value 0.05 assuming the null distribution is normally distributed). A high positive Z-score indicates that the raw data statistic is much greater than those obtained by chance (null hypothesis), and therefore is highly significant in a statistical sense. If the null distribution (of shuffle statistics) is non-normally distributed (Shapiro-Wilk test or Anderson-Darling test), we derived the Monte Carlo *P*-values from the sample distribution.

### Identification of hippocampal ripple candidate events during sleep and quiet wakefulness

During sleep, we focused on SWS epochs, which were primarily determined by the low EMG amplitude and high delta/theta power ratio in EEG activity (REM sleep is associated with low EMG, low delta/theta power ratio and high theta power). For screening the candidate events, we used hippocampal LFP ripple band (150–300 Hz) power combined with hippocampal multi-unit activity (threshold > mean + 3SD). We also imposed a minimum cell activation criterion (>6 or 10% of cell population, whichever is greater). Similar LFP and multi-unit activity criteria were also applied to QW periods, when the animal was in an immobile wake state (speed <2 cm s^−1^).

## Additional Information

**How to cite this article**: Chen, Z. *et al*. Uncovering representations of sleep-associated hippocampal ensemble spike activity. *Sci. Rep*. **6**, 32193; doi: 10.1038/srep32193 (2016).

## Supplementary Material

Supplementary Information

## Figures and Tables

**Table 1:** Summary statistics of ensemble recordings in the rat hippocampus.

Dataset	# Place cells	Rate (Hz) (mean ± SEM)	Recording, run time (min)	Recording environment
1	49	1.48 ± 0.45	24.3, 9.8	open field
2	37	1.32 ± 0.13	22.9, 12.3	open field
3	50	0.64 ± 0.07	28.9, 10.2	circular track
4a	77	1.11 ± 0.08	44.6, 25.4	circular track
4b	77	0.66 ± 0.05	480, N/A	rest/sleep box

Note that the mean firing rates of the same set of neurons reduce by nearly 50% from wake (4a) to sleep (4b).

**Table 2:** Comparison of median decoding error (mean ± SEM, *n* = 50 Monte Carlo runs) between Decode_wRF_ and Decode_woRF_ for Dataset 1.

*T*_0_ (bpe)	Decode_wRF_			Decode_woRF_		
	*ρ* = 0.3	*ρ* = 0.5	*ρ* = 0.8	*ρ* = 0.3	*ρ* = 0.5	*ρ* = 0.8
10	13.97 ± 0.07	10.49 ± 0.06	7.78 ± 0.02	12.56 ± 0.07	11.37 ± 0.06	10.61 ± 0.05
20	15.35 ± 0.12	11.03 ± 0.04	7.80 ± 0.01	12.39 ± 0.05	10.65 ± 0.04	9.71 ± 0.04
50	14.40 ± 0.08	10.87 ± 0.06	7.88 ± 0.02	11.15 ± 0.05	9.78 ± 0.05	8.51 ± 0.03
100	14.50 ± 0.09	10.78 ± 0.05	7.93 ± 0.02	11.49 ± 0.05	10.04 ± 0.04	8.84 ± 0.04
100*	15.97 ± 0.18	10.78 ± 0.09	7.93 ± 0.04	12.53 ± 0.16	11.51 ± 0.12	9.07 ± 0.18
	Δ = 50 ms	Δ = 150 ms	Δ = 250 ms	Δ = 50 ms	Δ = 150 ms	Δ = 250 ms
10	14.53 ± 0	8.58 ± 0	7.01 ± 0	16.21 ± 1.23	11.12 ± 0.32	10.52 ± 0.16
20	14.53 ± 0	8.58 ± 0	7.01 ± 0	16.35 ± 1.68	12.10 ± 0.31	10.02 ± 0.15
50	14.53 ± 0	8.58 ± 0	7.01 ± 0	16.68 ± 1.20	12.47 ± 0.32	10.17 ± 0.19
100	14.53 ± 0	8.58 ± 0	7.01 ± 0	15.40 ± 1.19	10.90 ± 0.34	8.38 ± 0.19

When varying *ρ*, we fixed Δ = 250 ms; when varying Δ, we fixed *ρ* = 1. Except for *T*_0_ = 100* bpe, the same active cells were used in each epoch.

**Table 3:** Summary statistics of candidate events (Dataset 4b).

State	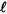	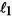	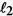	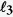	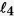	|*R*|	*Z*
pre-QW	338	165	87	15	15	0.56	1.83
			46	15	15	0.54	1.86
pre-SWS	984	471	256	33	31	0.60	1.93
			186	58	58	0.55	1.93
post-QW	1755	1015	627	100	100	0.62	2.05
			468	223	218	0.58	2.06
post-SWS	1519	764	440	62	60	0.59	2.02
			343	142	139	0.56	2.10

Notations: 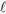: total epoch number; 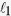: epoch number that had no less than 10% active cells; 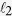: epoch number with |*R*| > 0.5; 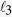: epoch number with *Z* > 1.65; 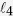: number of significant events based on three significance criteria. The last two columns show the median |*R*| and Z-score statistics derived from the 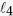 group. Two numbers in the last five columns show the results derived from Decode_wRF_ (top) and Decode_woRF_ (bottom), respectively.

## References

[b1] Buzsáki G. Memory consolidation during sleep: a neurophysiological perspective. J Sleep Res 7, 17–23 (1998). 9682189 10.1046/j.1365-2869.7.s1.3.x

[b2] Stickgold R. Sleep-dependent memory consolidation. Nature 437, 1272–1278 (2005). 16251952 10.1038/nature04286

[b3] Marshall L. & Born J. Contribution of sleep to hippocampus-dependent memory consolidation. Trends Cog Sci 11, 442–450 (2007). 10.1016/j.tics.2007.09.001 17905642

[b4] Wilson M. A. & McNaughton B. L. Reactivation of hippocampal ensemble memories during sleep. Science 265(5124), 676–679 (1994). 8036517 10.1126/science.8036517

[b5] Skaggs W. E. & McNaughton B. L. Replay of neuronal firing sequences in rat hippocampus during sleep following spatial experience. Science 271, 1870–1873 (1996). 8596957 10.1126/science.271.5257.1870

[b6] Kudrimoti H. S., Barnes C. A. & McNaughton B. L. Reactivation of hippocampal cell assemblies: Effects of behavioral state, experience, and EEG dynamics. J Neurosci 19(10), 4090–4101 (1999). 10234037 10.1523/JNEUROSCI.19-10-04090.1999 PMC6782694

[b7] Louie K. & Wilson M. A. Temporally structured replay of awake hippocampal ensemble activity during rapid eye movement sleep. Neuron 29, 145–156 (2001). 11182087 10.1016/s0896-6273(01)00186-6

[b8] Lee A. K. & Wilson M. A. Memory of sequential experience in the hippocampus during slow wave sleep. Neuron 36(6), 1183–1194 (2002). 12495631 10.1016/s0896-6273(02)01096-6

[b9] Lee A. K. & Wilson M. A. A combinatorial method for analyzing sequential firing patterns involving an arbitrary number of neurons based on relative time order. J Neurophysiol 92, 2555–2573 (2004). 15212425 10.1152/jn.01030.2003

[b10] Ji D. & Wilson M. A. Coordinated memory replay in the visual cortex and hippocampus during sleep. Nat Neurosci 10(1), 100–107 (2007). 17173043 10.1038/nn1825

[b11] OÕKeefe J. & Dostrovsky J. The hippocampus as a spatial map: Preliminary evidence from unit activity in the freely-moving rat. Brain Res 34, 171–175 (1971). 5124915 10.1016/0006-8993(71)90358-1

[b12] Sirota A., Csicsvari J., Buhl D. & Buzsáki G. Communication between neocortex and hippocampus during sleep in rodents. Proc Natl Acad Sci USA 100, 2065–2069 (2003). 12576550 10.1073/pnas.0437938100 PMC149959

[b13] Dragoi G. & Tonegawa S. Preplay of future place cell sequences by hippocampal cellular assemblies. Nature 469(7330), 397–401 (2011). 21179088 10.1038/nature09633 PMC3104398

[b14] Dragoi G. & Tonegawa S. Distinct preplay of multiple novel spatial experiences in the rat. Proc Natl Acad Sci USA 110(22), 9100–9105 (2013). 23671088 10.1073/pnas.1306031110 PMC3670374

[b15] Nádasdy Z., Hirase H., Czurkó A., Csicsvari J. & Buzsáki G. Replay and time compression of recurring spike sequences in the hippocampus. J Neurosci 19(12), 9497–9507 (1999). 10531452 10.1523/JNEUROSCI.19-21-09497.1999 PMC6782894

[b16] Pfeiffer B. E. & Foster D. J. Hippocampal place-cell sequences depict future paths to remembered goals. Nature 497, 74–79 (2013). 23594744 10.1038/nature12112 PMC3990408

[b17] Wu X. & Foster D. J. Hippocampal replay captures the unique topological structure of a novel environment. J Neurosci 34(19), 6459–6469 (2014). 24806672 10.1523/JNEUROSCI.3414-13.2014 PMC4012305

[b18] Silva D., Feng T. & Foster D. J. Trajectory events across hippocampal place cells require previous experience. Nat Neurosci 18, 1772–1779 (2015). 26502260 10.1038/nn.4151 PMC6095134

[b19] Kay K. . A hippocampal network for spatial coding during immobility and sleep. Nature 531, 185–190 (2016). 26934224 10.1038/nature17144 PMC5037107

[b20] Smith A. C., Nguyen V. K., Karlsson M. P., Frank L. M. & Smith P. Probability of repeating patterns in simultaneous neural data. Neural Comput 22, 2522–2536 (2010). 20608872 10.1162/NECO_a_00020

[b21] Brown E. N., Frank L. M., Tang D., Quirk M. C. & Wilson M. A. A statistical paradigm for neural spike train decoding applied to position prediction from ensemble firing patterns of rat hippocampal place cells. J Neurosci 18(18), 7411–7425 (1998). 9736661 10.1523/JNEUROSCI.18-18-07411.1998 PMC6793233

[b22] Zhang K., Ginzburg I., McNaughton B. L. & Sejnowski T. J. Interpreting neuronal population activity by reconstruction: unified framework with application to hippocampal place cells. J Neurophysiol 79(2), 1017–1044 (1998). 9463459 10.1152/jn.1998.79.2.1017

[b23] Davidson T. J., Kloosterman F. & Wilson M. A. Hippocampal replay of extended experience. Neuron 63(4), 497–507 (2009). 19709631 10.1016/j.neuron.2009.07.027 PMC4364032

[b24] Kloosterman F., Layton S., Chen Z. & Wilson M. A. Bayesian decoding of unsorted spikes in the rat hippocampus. J Neurophysiol 111(1), 217–227 (2014). 24089403 10.1152/jn.01046.2012 PMC3921373

[b25] Buzsáki G. & Mizuseki K. The log-dynamic brain: how skewed distributions affect network operations. Nat Rev Neurosci, 26, 88–95 (2014). 10.1038/nrn3687 PMC4051294 24569488

[b26] Leutgeb S. . Independent codes for spatial and episodic memory in hippocampal neuronal ensembles. Science 309, 619–623 (2005). 16040709 10.1126/science.1114037

[b27] Hirase H., Leinekugel X., Czurko A., Csicsvari J. & Buzsáki G. Firing rates of hippocampal neurons are preserved during subsequent sleep episodes and modified by novel awake experiences. Proc Natl Acad Sci USA 98, 9386–9390 (2001). 11470910 10.1073/pnas.161274398 PMC55430

[b28] Chen Z., Kloosterman F., Brown E. N. & Wilson M. A. Uncovering hidden spatial topology represented by hippocampal population neuronal codes. J Comput Neurosci 33(2), 227–255 (2012). 22307459 10.1007/s10827-012-0384-x PMC3974406

[b29] Chen Z., Gomperts S., Yamamoto J. & Wilson M. A. Neural representation of spatial topology in the rodent hippocampus. Neural Comput 26(1), 1–39 (2014). 24102128 10.1162/NECO_a_00538 PMC3967246

[b30] Linderman S. W., Johnson M. J., Wilson M. A. & Chen Z. A Bayesian nonparametric approach to uncovering rat hippocampal population codes during spatial navigation. J Neurosci Methods 236, 36–47 (2016). 26854398 10.1016/j.jneumeth.2016.01.022 PMC4801699

[b31] Grosmark A. D. & Buzsáki G. Diversity in neural firing dynamics supports both rigid and learned hippocampal sequences Science 351(6280), 1440–1443 (2016). 27013730 10.1126/science.aad1935 PMC4919122

[b32] Diba K. & Buzsáki G. Forward and reverse hippocampal place-cell sequences during ripples. Nat Neurosci 10(10), 100–107 (2007). 17828259 10.1038/nn1961 PMC2039924

[b33] Carr M. F., Jadhav S. P. & Frank L. M. Hippocampal replay in the awake state: a potential physiological substrate of memory consolidation and retrieval. Nat Neurosci. 14, 147–153 (2011). 21270783 10.1038/nn.2732 PMC3215304

[b34] Pfeiffer B. E. & Foster D. J. Autoassociative dynamics in the generation of sequences of hippocampal place cells. Science 349, 180–183 (2015). 26160946 10.1126/science.aaa9633

[b35] Bendor D. & Wilson M. A. Biasing the content of hippocampal replay during sleep. Nat Neurosci 15, 1439–1444 (2012). 22941111 10.1038/nn.3203 PMC4354843

[b36] Roumis D. K. & Frank L. M. Hippocampal sharp-wave ripples in waking and sleep states. Curr Opin Neurobiol 35, 6–12 (2015). 26011627 10.1016/j.conb.2015.05.001 PMC4641767

[b37] Jacobs A. L. . Ruling out and ruling in neural codes. Proc Natl Acad Sci USA 106(14), 5936–5941 (2009). 19297621 10.1073/pnas.0900573106 PMC2657589

[b38] Pillow J. W., Ahmadian Y. & Paninski L. Model-based decoding, information estimation, and change-point detection techniques for multineuron spike trains. Neural Comput 23(1), 1–45 (2009). 20964538 10.1162/NECO_a_00058

[b39] Giusti C., Pastalkova E., Curto C. & Itskov V. Clique topology reveals intrinsic geometric structure in neural correlations. Proc Nat Acad Sci USA 112(44), 13455–13460 (2015). 26487684 10.1073/pnas.1506407112 PMC4640785

[b40] Peyrache A., Khamassi M., Benchenane K., Wiener S. & Battaglia F. Replay of rule-learning related neural patterns in the prefrontal cortex during sleep. Nat Neurosci 12(7), 919–926 (2009). 19483687 10.1038/nn.2337

[b41] Peyrache A., Lacroix M. M., Petersen P. C. & Buzsáki G. Internally organized mechanisms of the head direction sense. Nat Neurosci 18, 569–575 (2015). 25730672 10.1038/nn.3968 PMC4376557

[b42] Gulati T., Ramanathan D. S., Wong C. C. & Ganguly K. Reactivation of emergent task-related ensembles during slow-wave sleep after neuroprosthetic learning. Nat Neurosci 17(8), 1107–1113 (2014). 24997761 10.1038/nn.3759 PMC5568667

[b43] Skaggs W. E., McNaughton B. L. & Gothard K. M. An information-theoretic approach to deciphering the hippocampal code. Advances in Neural Information Processing System 5 (pp. 1030–1037). San Francisco, CA: Morgan Kaufman Publisher (1993).

