# Supplementary material for: Uncovering representations of sleep-associated hippocampal ensemble spike activity
Source: Sci Rep. 2016 Aug 30;6:32193. doi: 10.1038/srep32193 (PMC5004124; doi:10.1038/srep32193)
Supplement: Supplementary Information [file srep32193-s1.pdf]

Uncovering representations of sleep-associated hippocampal ensemble spike activity

Zhe Chen, Andres D. Grosmark, Hector Penagos, & Matthew A. Wilson

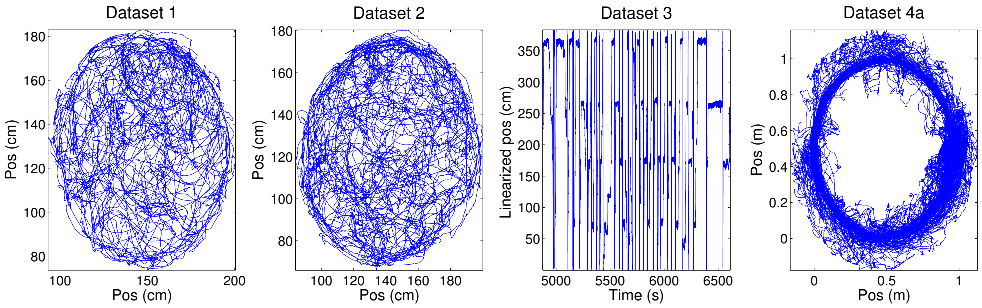

Figure S1: Spatial trajectories during animal's run behavior from four different datasets.

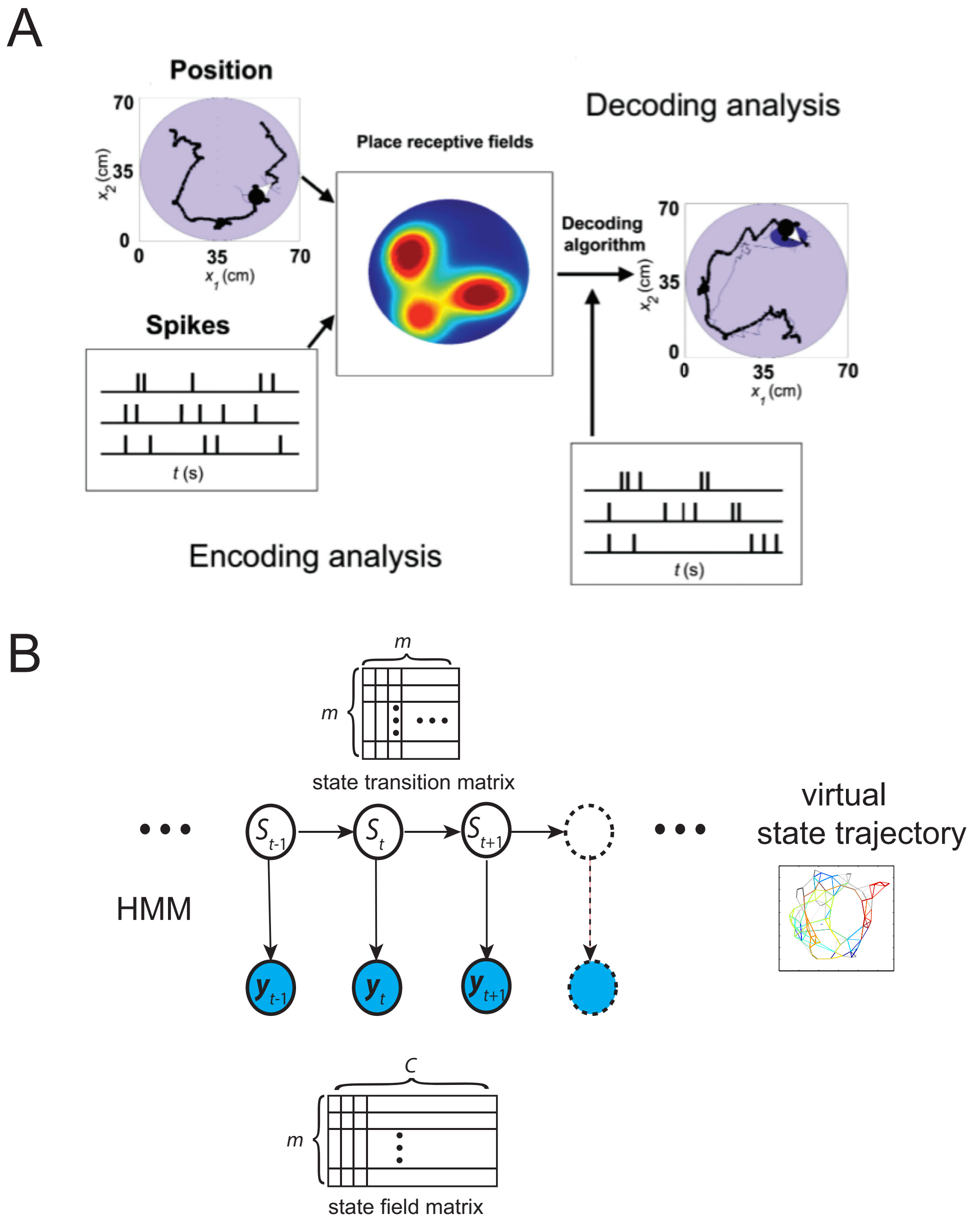

Figure S2: **Schematic illustration of two rat hippocampal population-decoding methods.** (*A*) $\text{Decode}_{\text{wRF}}$: during the encoding phase, animal's spatial position is measured and each neuron's receptive field is estimated; during the decoding phase, the receptive fields are used to evaluate the data likelihood (adapted from ref. 44, with permission). The thick curve denotes the animal's past trajectory, and the triangle represents the animal's position. The think curve denotes the posterior estimate of trajectory, and the blue circle represents the confidence interval of the estimate. (*B*) $\text{Decode}_{\text{woRF}}$: the animal's behavior is inaccessible, and the latent discrete variable ("virtual position") S_t follows a Markovian process and forms a hidden Markov model (HMM), illustrated by a graphic model (where the open and filled circles represent respective latent and observed random variables, and the arrow indicates statistical dependence between random variables). The state transition follows a stationary $m \times m$ state transition matrix, and the state trajectories are associated with consistent hippocampal ensemble spiking patterns, as characterized by an $m \times C$ state field matrix (bottom), where C denotes the number of neurons in population. The inferred state sequences correspond to a virtual state trajectory, that is navigating in a color-coded topological graph (ref. 29), where the warm/cold color represents the direction of time evolution.

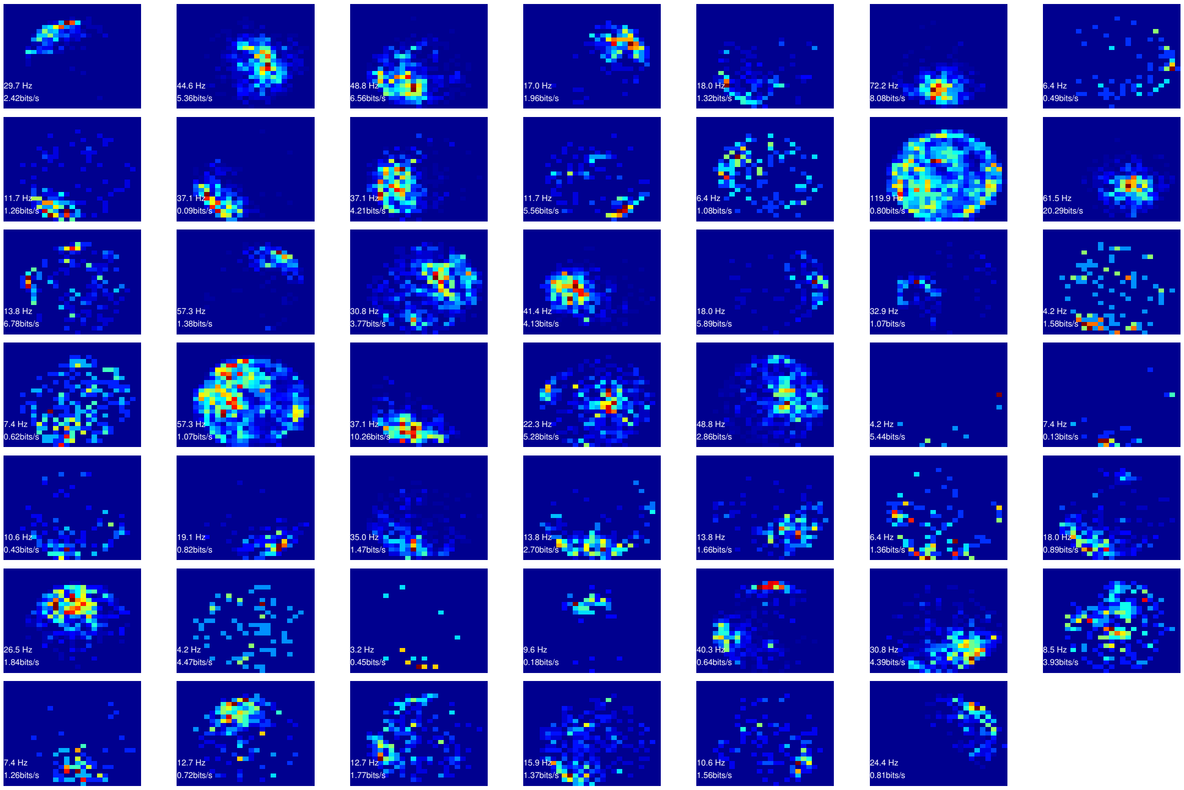

Figure S3: **Place receptive fields used in** $\text{Decode}_{\text{wRF}}$. Visualization of 48 place receptive fields (bin size: 5×5 cm^2) of rat hippocampal neurons (Dataset 1). Numbers in each panel indicate the peak firing rate (unit: Hz or spikes/(s·bin)) and spatial information rate (bits/s), and warm color represents a high firing rate. Note that there was a large variability in both peaking firing rate and spatial coverage across all neurons. The mean firing rate and information rate of these neurons are positively correlated (Pearson's correlation: 0.93).

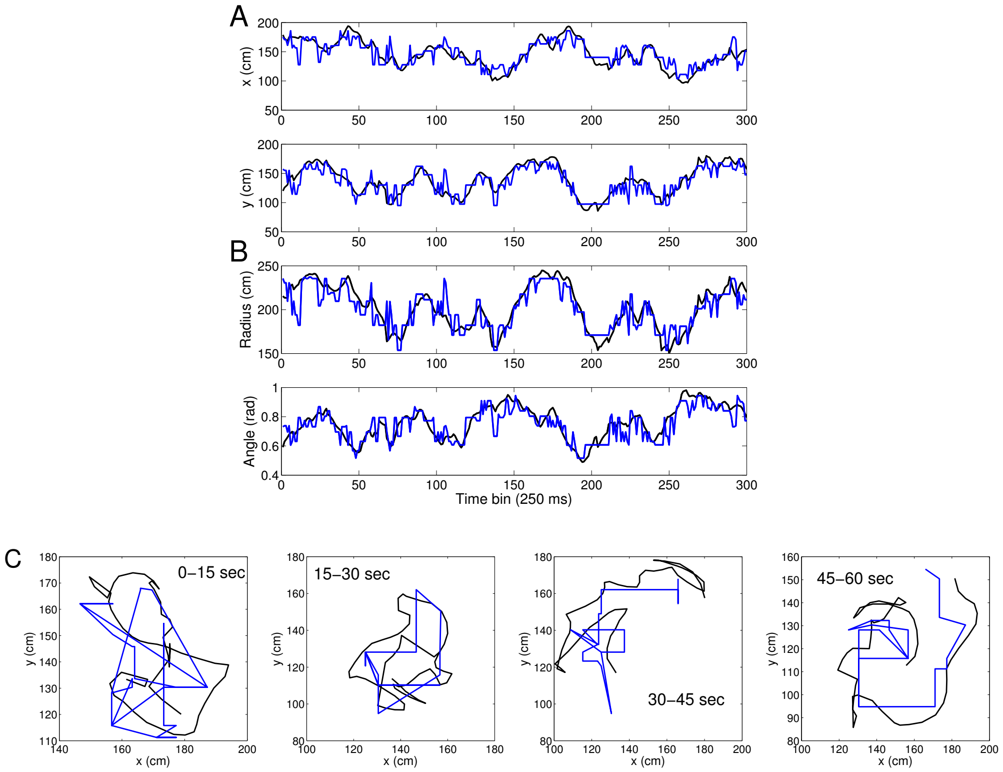

Figure S4: **Population decoding derived from** $\text{Decode}_{\text{woRF}}$ **without place receptive fields.** Snapshot illustration for population decoding performance of $\text{Decode}_{\text{woRF}}$ in a two-dimensional open field (Dataset 1, using hippocampal ensemble spike data alone). Black and blue curves denote the animal's position and decoded state trajectories, respectively. Average median error 8.37 cm, mean error 9.44 cm for the complete dataset. (*A*) Cartesian coordinate representation. (*B*) Polar coordinate representation. (*C*) Two-dimensional representation in four consecutive windows: 0-15 sec, 15-30 sec, 30-45 sec, and 45-60 sec. Note that the temporal smoothness of two-dimensional trajectories was lost due to lack of precision at a fine timescale.

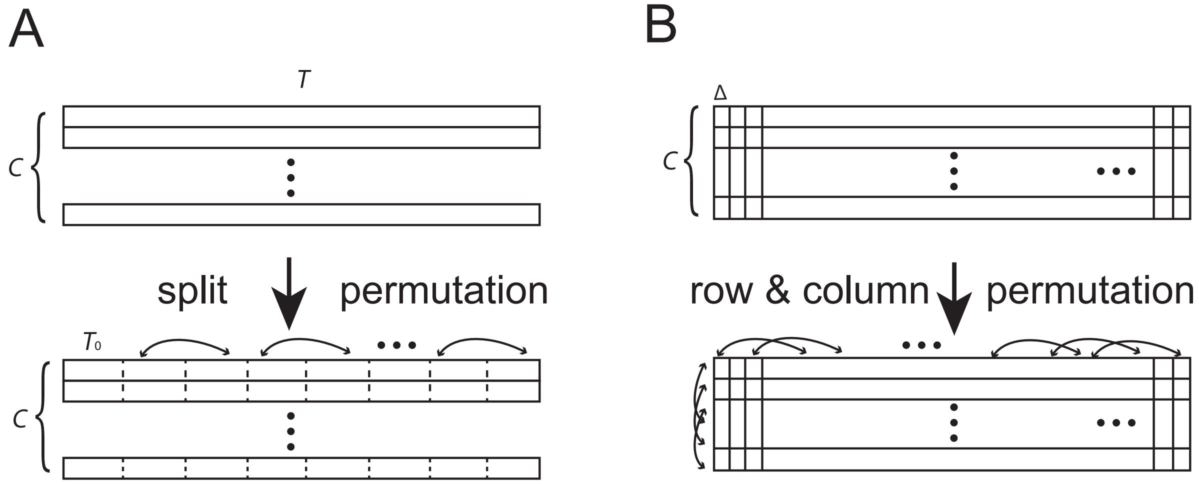

Figure S5: **Manipulation of synthetic sleep and shuffled data.** Cartoon illustrations of synthetic sleep spike data generation (A) and shuffled spike data generation (B). The ensemble spike trains are represented as a C-by-T matrix, with a bin size of Δ. The complete data are split into ℓ epochs, and each epoch is represented as a C-by-T_0 matrix (where $T_0 = \frac{T}{\ell}$ bins per epoch). The temporal direction of each epoch is either preserved or reversed (with equal probability). Except for the possible change in flipping direction, the spatiotemporal entries of original C-by-T_0 matrix remain unchanged.

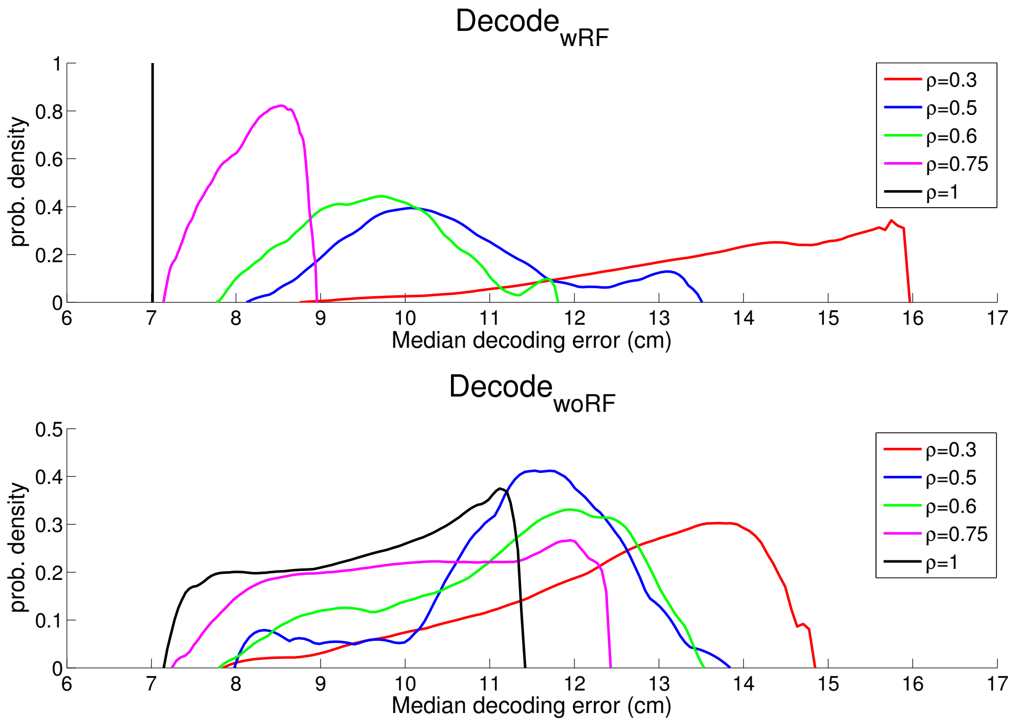

Figure S6: **Evolution of median decoding error distributions with varying ρ.** Comparison of median decoding error distributions between Decode$_{\mathrm{wRF}}$ (with 5×5 cm^2 receptive field) and Decode$_{\mathrm{woRF}}$ for varying active cell ratio ρ (Dataset 1, $T_0 = 10$ bpe). Note that the error support is gradually shifted to the right with a decreasing ρ. Also note that strong skewness of decoding error distribution in both panels, especially for a smaller ρ value, suggesting a *non-even* neuronal contribution in population representation (i.e., different choices of cell subsampling with an identical ρ could have varying effects on decoding accuracy). An even contribution among neurons would imply a symmetric distribution in decoding error.

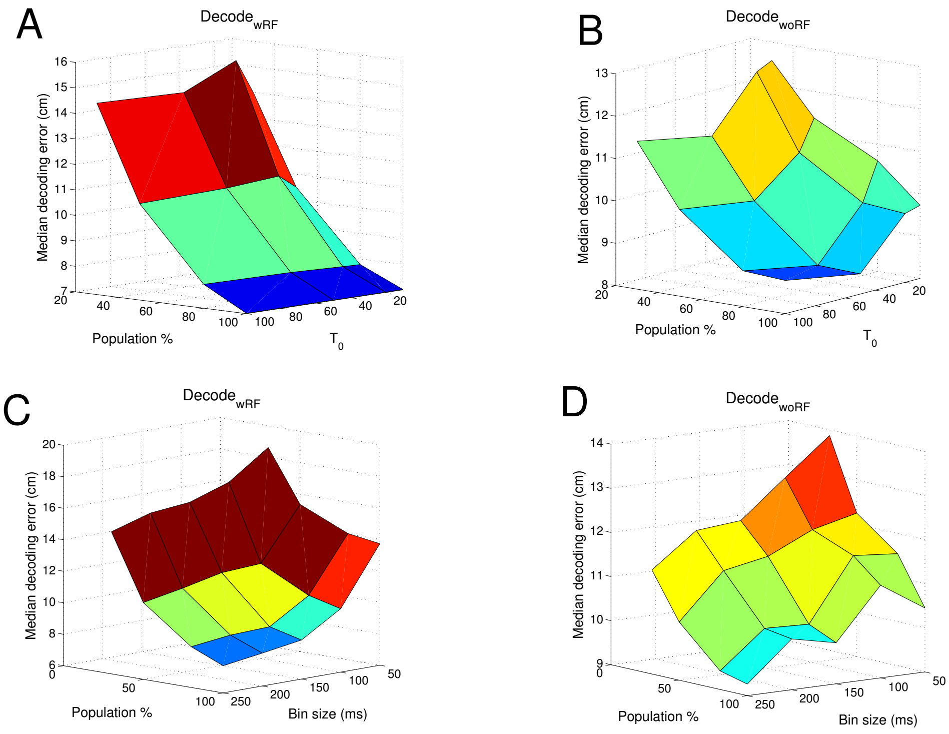

Figure S7: **Impact of conjugative factors on the decoding error.** Comparison of averaged median decoding error surface between Decode$_{\mathrm{wRF}}$ (with 5×5 cm^2 receptive field) and Decode$_{\mathrm{woRF}}$ by jointly varying population % and T_0 (panels *A*,*B*, Dataset 1, fixed $\Delta = 250$ ms) and by jointly varying population % and temporal bin size (panels *C*,*D*, Dataset 2, fixed $T_0 = 10$ bpe).

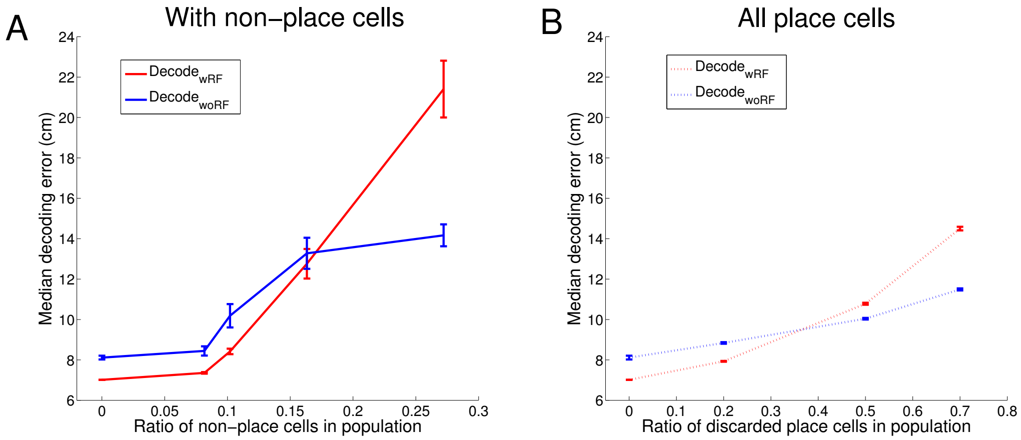

Figure S8: **Impact of non-place cells in cell population on the decoding error.** Comparison of median decoding error (mean±SEM, over 50 Monte Carlo runs) between two population decoding methods in the presence of non-place cells (*A*, solid lines) or all-place cells (*B*, dotted lines) under the same configuration (Dataset 1, $C = 49$, $T_0 =$ 100 bpe). (*A*) The percentages of non-place cells are $0/49, 4/49, 4/(49 * 0.8), 4/(49 * 0.5), 4/(49 * 0.3)$, from the left to right x-axis, respectively, where the numerator and denominator ($C * \rho$) denote the numbers of non-place cells and total cells, respectively. The decoding accuracy degrades as the result of introducing non-place cells or decreasing SNR. (*B*) The number of all-place cells in use are $49, 49 * 0.8, 49 * 0.5, 49 * 0.3$ from the left to right x-axis, respectively. In both conditions, $\text{Decode}_{\text{woRF}}$ (red) performed better than $\text{Decode}_{\text{wRF}}$ (blue) in the case of very low SNR.

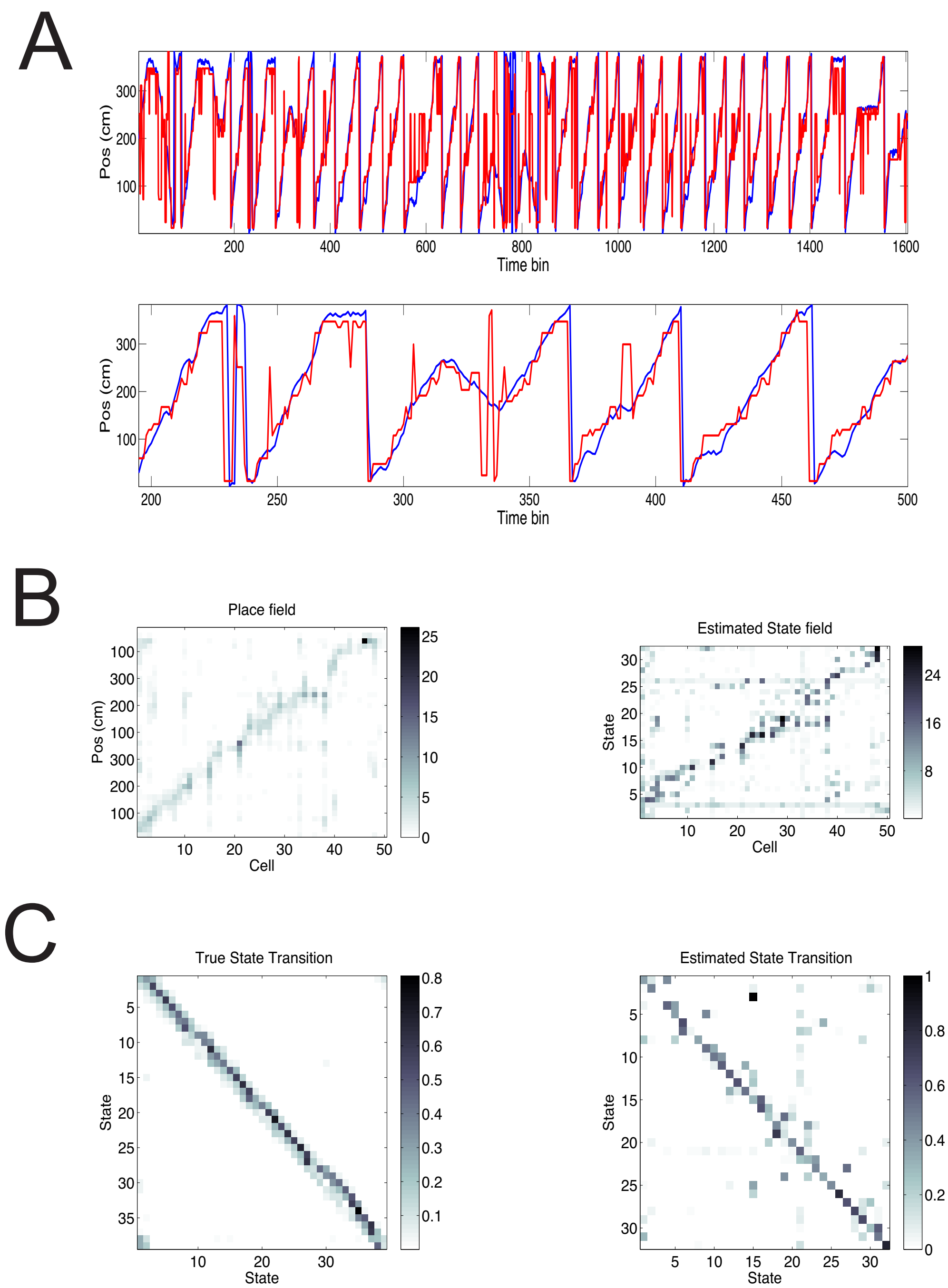

Figure S9: **Illustration of decoding results (Dataset 3) derived from** $\mathrm{Decode}_{\mathrm{woRF}}$ **without place receptive fields.** (*A*) Animal's linearized spatial position (blue) versus remapped state trajectory derived from $\mathrm{Decode}_{\mathrm{woRF}}$ (red), indicating a high degree of correlation (Pearson's correlation: 0.78). Bottom panel shows a zoom-in period of the top panel. Time bin size $\Delta = 250$ ms. (*B*) *Left*: Hippocampal place receptive fields estimated from behavior. *Right*: Inferred state fields from $\mathrm{Decode}_{\mathrm{woRF}}$ based on ensemble spike data alone. (*C*) *Left*: Animal's actual space transition matrix estimated from behavior. *Right*: Inferred state transition matrix from $\mathrm{Decode}_{\mathrm{woRF}}$ based on ensemble spike data alone.

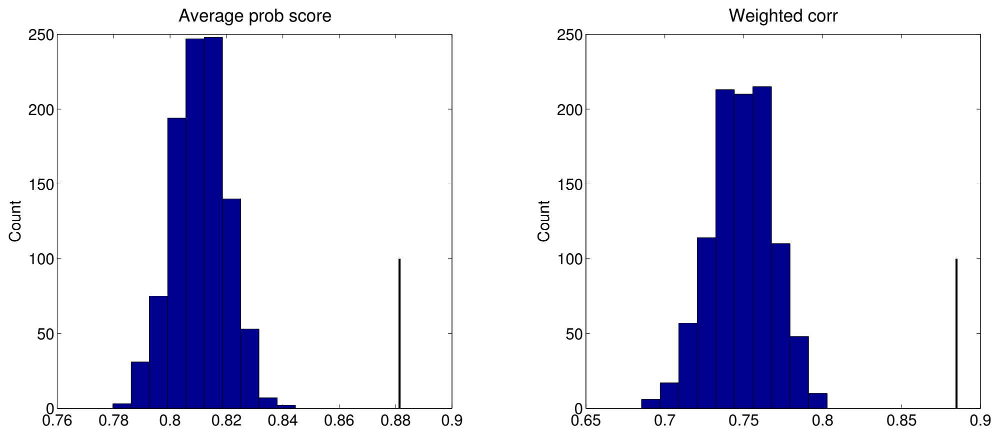

Figure S10: **Comparison of shuffled statistics with raw statistics.** Results of shuffled data analyses on the average MAP probability score (*Left*) and weighted correlation (*Right*), derived from $\text{Decode}_{\text{woRF}}$. The histograms were generated from the shuffled statistics, whereas the raw statistic was showed in vertical line. The Z-score values for these plots are 6.43 and 5.68, respectively, indicating a high level of statistical significance.

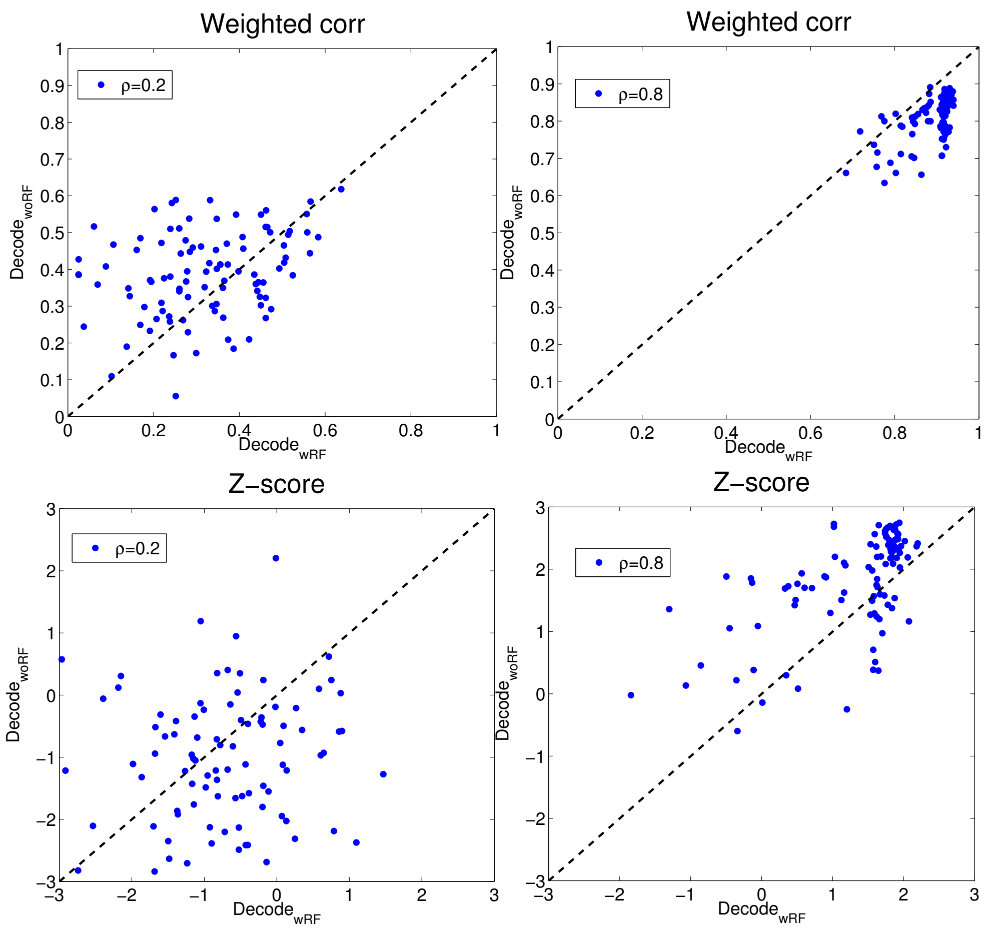

Figure S11: **Detection power improves with increasing number of neurons.** Comparison of weighted correlation and associated Z-score statistics between Decode$_{\text{woRF}}$ and Decode$_{\text{woRF}}$ (Dataset 3) between a low ($\rho = 0.2$) and high ($\rho = 0.8$) ratio of active neurons. Each point represents a sampled result from a Monte Carlo simulation. Both population decoding methods achieved high (>0.5) weighted correlation statistics for $\rho = 0.8$, with a better performance in Decode$_{\text{wRF}}$. In terms of Z-score, the detection power of these two methods were not significantly different for $\rho = 0.2$ ($P > 0.05$, paired t-test); Decode$_{\text{woRF}}$ had a higher detection power than Decode$_{\text{wRF}}$ for $\rho = 0.8$ ($P < 10^{-4}$, paired t-test).

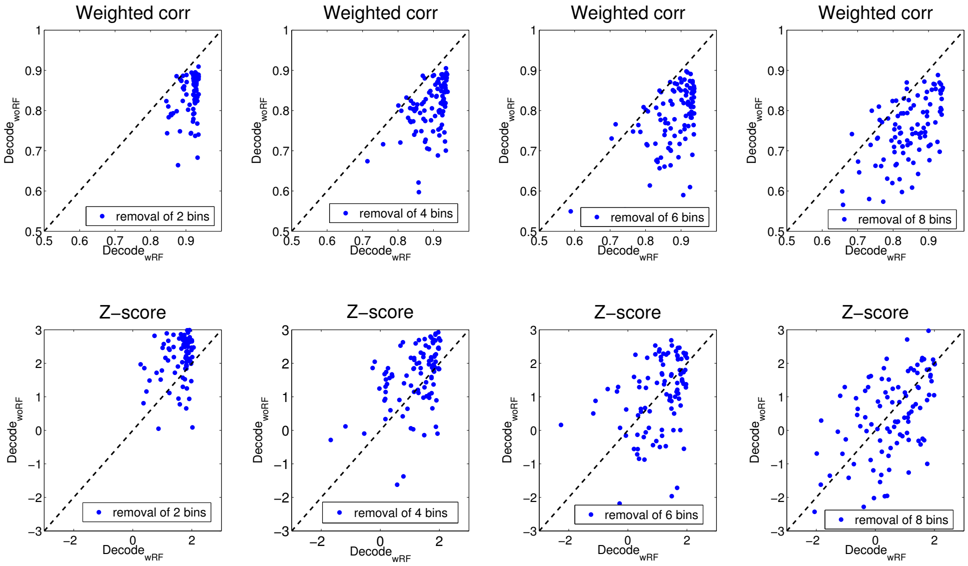

Figure S12: **Detection power degrades by removing spikes or temporal bins.** Comparison of weighted correlation and associated Z-score statistics between Decode$_{\mathrm{wRF}}$ and Decode$_{\mathrm{woRF}}$ (Dataset 3) by randomly removing a few number of temporal bins. Although Decode$_{\mathrm{wRF}}$ obtained higher weighted correlation statistics, Decode$_{\mathrm{woRF}}$ had higher Z-score statistics than Decode$_{\mathrm{wRF}}$ in the first two columns ($P < 10^{-4}$, paired t-test).

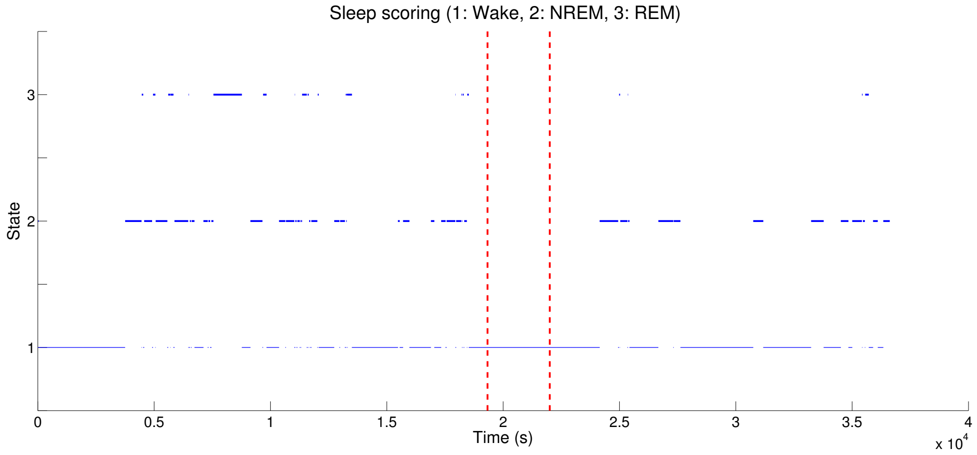

Figure S13: **Brain state classification (Datasets 4a and 4b).** Vertical dashed lines mark the onset and offset of animal's run behavior on the circular track.

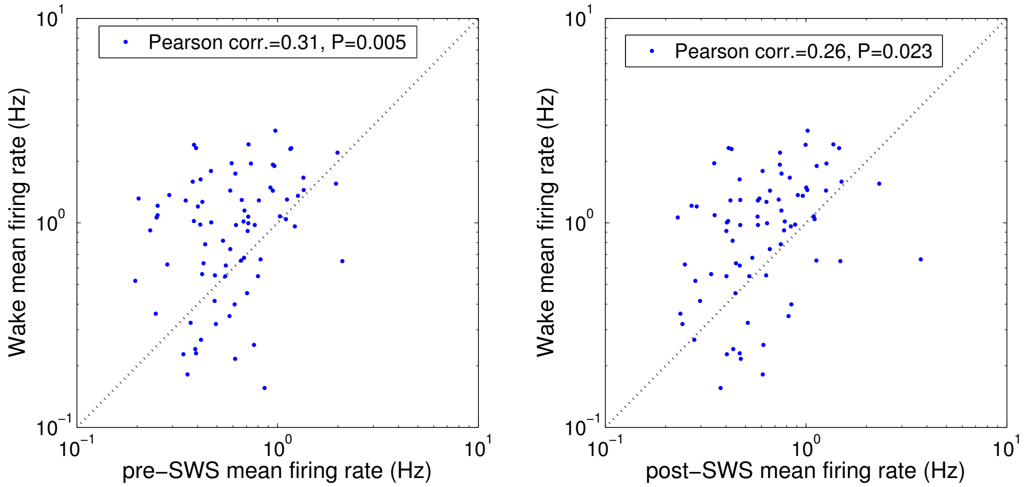

Figure S14: **Comparison in mean firing rate of hippocampal neurons between different brain states (Datasets 4a and 4b).** *Left*: pre-SWS vs. wake. *Right:* post-SWS vs. wake. In both comparison, mean firing rates between different brain states are positively correlated. The mean firing rate in wake was significantly greater than in pre-SWS (Wilcoxon signed rank test, $P = 1.19 \times 10^{-5}$) and than in post-SWS (Wilcoxon signed rank test, $P = 1.23 \times 10^{-5}$).

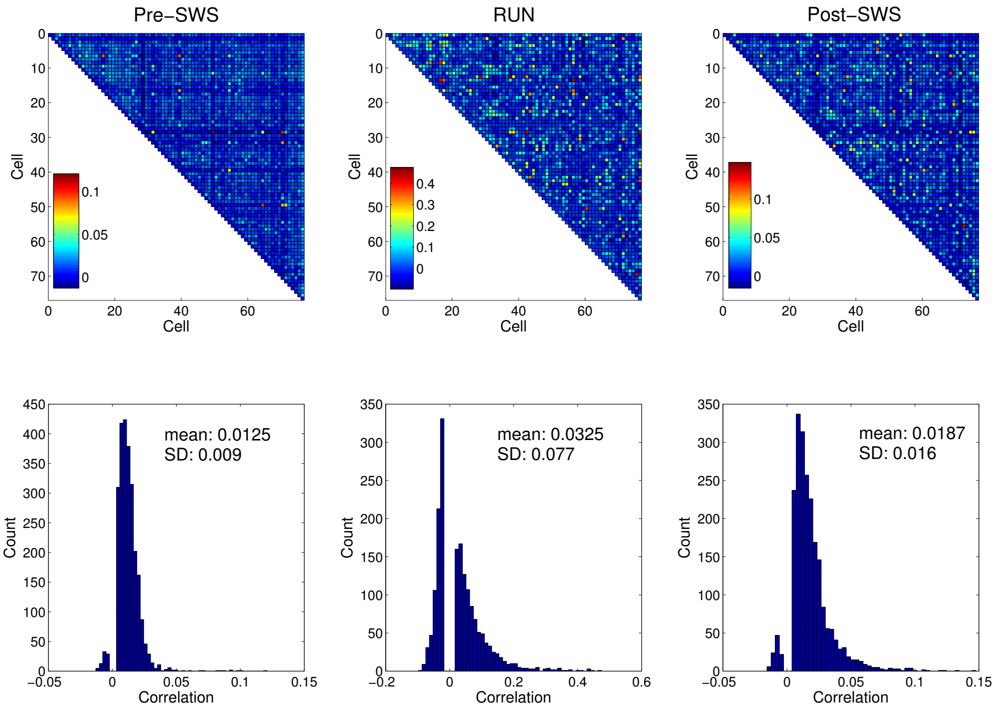

Figure S15: **Correlation statistics of hippocampal population across states.** Comparison of pairwise correlation of hippocampal neuronal spike counts between different brain states (pre-SWS vs. RUN vs. post-SWS; Datasets 4a and 4b). In top three panels, cells were arranged in the same order. Correlation without statistical significance was marked as zero. In the bottom panels, the histograms of nonzero correlation values are shown with reported mean and SD statistics. In both pre-SWS and post-SWS, the population spike trains were binned with 20 ms; whereas in RUN, the population spike trains were binned with 250 ms.

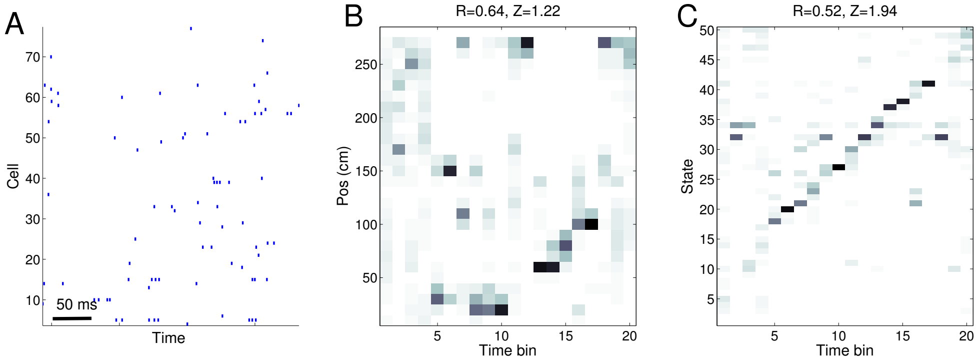

Figure S16: **Examples of post-SWS spike data analysis.** (*A*) Spike raster of a post-SWS candidate event. (*B*) Posterior probability map (position bin × time bin: 29×20) obtained from $\text{Decode}_{\text{wRF}}$. (*C*) Posterior probability map (state × time bin: 50×20) obtained from $\text{Decode}_{\text{woRF}}$. Note that the discontinuity of "linear fit" in panel *B*. In contrast, panel *C* has a finer resolution in the vertical axis, as the state dimensionality was determined automatically from the data. In this example, the Z-score of weighted correlation R derived from $\text{Decode}_{\text{wRF}}$ did not meet the significance criterion, even it obtained a greater R value.

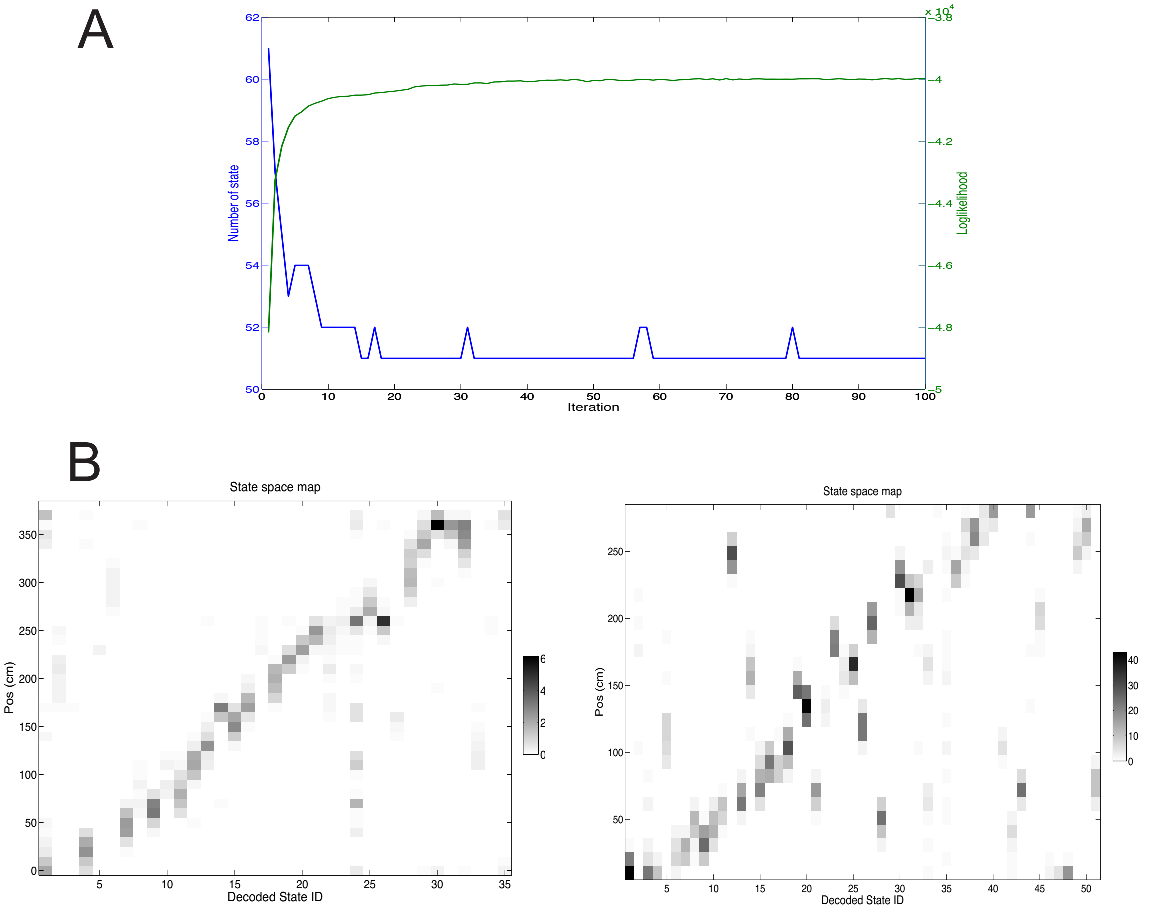

Figure S17: **Algorithmic performance of** $\text{Decode}_{\text{woRF}}$**.** (*A*) Convergence curves of MCMC inference for $\text{Decode}_{\text{woRF}}$ (Dataset 4a). The number of latent states (blue) was automatically estimated from the Bayesian inference procedure (with a finally converged estimate of $m = 51$ states). The log likelihood (red) increased monotonically. (*B*) State space maps (*Left*: Dataset 3; *Right*: Dataset 4a) derived from $\text{Decode}_{\text{woRF}}$. A one-to-one correspondence would indicate a perfect mapping between the latent state and spatial position.

References

44. Brown EN, Kass RE, Mitra PP (2004). Multiple neural spike train data analysis: state-of-the-art and future challenges. *Nat Neurosci* 7(5): 456-461.
